# Implications of Sphingolipids on Aging and Age-Related Diseases

**DOI:** 10.3389/fragi.2021.797320

**Published:** 2022-03-03

**Authors:** Shengxin Li, Hyun-Eui Kim

**Affiliations:** ^1^ Department of Integrative Biology and Pharmacology, McGovern Medical School, University of Texas Health Science Center at Houston, TX, United States; ^2^ Graduate School of Biomedical Sciences, University of Texas MD Anderson Cancer Center, Houston, TX, United States

**Keywords:** aging, lipids, sphingolipids, ceramide, age-related diseases, hallmarks of aging

## Abstract

Aging is a process leading to a progressive loss of physiological integrity and homeostasis, and a primary risk factor for many late-onset chronic diseases. The mechanisms underlying aging have long piqued the curiosity of scientists. However, the idea that aging is a biological process susceptible to genetic manipulation was not well established until the discovery that the inhibition of insulin/IGF-1 signaling extended the lifespan of *C. elegans*. Although aging is a complex multisystem process, López-Otín et al*.* described aging in reference to nine hallmarks of aging. These nine hallmarks include: genomic instability, telomere attrition, epigenetic alterations, loss of proteostasis, deregulated nutrient sensing, mitochondrial dysfunction, cellular senescence, stem cell exhaustion, and altered intercellular communication. Due to recent advances in lipidomic, investigation into the role of lipids in biological aging has intensified, particularly the role of sphingolipids (SL). SLs are a diverse group of lipids originating from the Endoplasmic Reticulum (ER) and can be modified to create a vastly diverse group of bioactive metabolites that regulate almost every major cellular process, including cell cycle regulation, senescence, proliferation, and apoptosis. Although SL biology reaches all nine hallmarks of aging, its contribution to each hallmark is disproportionate. In this review, we will discuss in detail the major contributions of SLs to the hallmarks of aging and age-related diseases while also summarizing the importance of their other minor but integral contributions.

## Introduction

Life expectancy has significantly increased over the last two hundred years due to the increase in quality of water, hygiene, and modern medicine. In recent decades, research in aging has grown in popularity due to the discovery of the notion that aging, like many other biological functions, can be subjected to genetic and environmental interventions (Kenyon, 2010). Several hallmarks of aging have been identified, highlighting the importance of several key physiological processes in aging. These hallmarks include deregulated nutrient sensing, cellular senescence, loss of proteostasis, genetic instability, altered cellular communication, mitochondria dysfunction, and stem cell exhaustion (López-Otín et al., 2013). One field that has intensified over the years is the study of lipids and their roles in aging.

Lipids are a large group of diverse macromolecules involved in key biological functions including serving as key structural components of cell and organelle membranes, a source of stored metabolic energy and signaling intermediates in various signaling pathways. There is a diverse structural variety among lipids, encompassing fatty acid and their derivatives to sterol-containing metabolites. Lipids are broken down into eight categories as defined by the International Lipid Classification and Nomenclature Committee (ILCNC). These include fatty acyls (FA), glycerolipids (GL), glycerophospholipids (GP), sphingolipids (SL), sterol lipids (ST), phenol lipids (PR), saccharolipids (SaL) and polyketides (PK) (Fahy et al., 2009). Studies have unveiled unique relationships between lipid profiles and aging in many of the model organisms including humans (Bustos and Partridge, 2017).

Before the early 1990s, the field of lipid studies was rooted exclusively in their functions in cellular structure and energy metabolism. However, in 1992, the discovery that diacylglycerol (DAG) directly activates protein kinase C (PKC) (Nishizuka, 1992) provided the spark that was needed to ignite the field of bioactive lipids. One class of bioactive lipids that have been garnering extensive interest in the past 20 years are the sphingolipids (SL). SLs are lipids that have a sphingosine backbone with a polar head group and nonpolar fatty acid tail. They represent a major class of lipids that have important implications in membrane biology, and as research interest increased and technology advanced, their roles as bioactive lipids have also been extensively studied, elucidating key regulatory functions in organismal development and homeostasis (Sheng, 2018). Unfortunately, SL research has been extremely difficult due to its diversity in structure and the complex regulatory pathways, however, an emerging body of evidence has emphasized the importance of SLs in aging. In this review, we will summarize the SL synthesis pathways and their regulatory elements followed by the contribution of SLs to the hallmarks of aging and age-related diseases. Our goal here is to bring together discoveries from different fields of SL research and to provide a comprehensive view of the evidence for the integral role SL plays in the physiological processes of aging.

### Sphingolipid Biosynthesis Pathway

**FIGURE 1:**
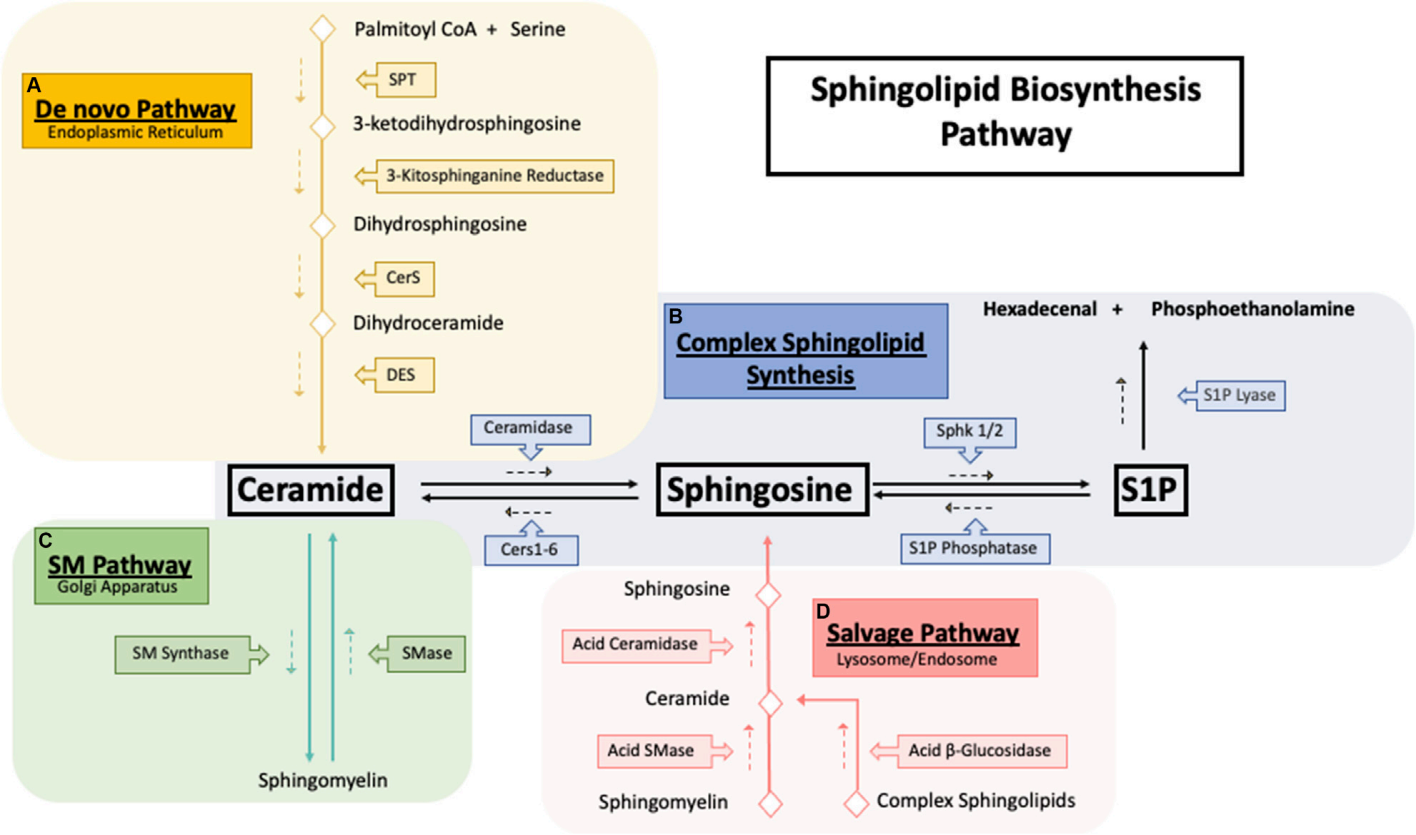
Sphingolipid synthesis pathways. Sphingolipid biosynthesis pathways. **(A)** The *De novo* sphingolipid pathway occurs in the endoplasmic reticulum (ER) where the condensation of serine and palmitoyl CoA by serine palmitoyltransferase (SPT) generates the backbone required for the synthesis of ceramide. Ceramide generated by the de novo pathway is then shuttled to the golgi apparatus **(B)** to be used as building blocks for the synthesis of sphingomyelin and other complex sphingolipids. **(C)** Exit from the sphingolipid synthesis pathways is initiated through S1P lyase where S1P cleavage results in hexadecenal and phosphoethanolamine which is further metabolized into palmitoyl CoA. **(D)** Catabolism of sphingolipids occurs in the lysosome where complex sphingolipids is broken down into ceramide which then is ultimately deacylated to sphingosine by acid ceramidase. Sphingosine then exits the lysosome and is synthesized back into ceramide to be further used where needed.

Sphingolipids are a vastly diverse class of lipids essential for proper cellular function regulation, particularly structure and signaling. The *de novo* sphingolipid biosynthesis pathway, through which all sphingolipids are built from nonsphingolipid precursors, begins in the ER (Figure 1A).

First, serine palmitoyltransferase (SPT) catalyzes the condensation of serine and palmitoyl CoA into 3-ketosphinganine followed by reduction into sphinganine by 3-kitosphinganine reductase (Perry, 2002). Next (dihydro)ceramide synthase, commonly known as ceramide synthase (CerS 1-6) converts sphinganine into dihydroceramide (Pewzner-Jung et al., 2006). Based on current evidence, each CerS likely prefers a distinct, but overlapping acyl CoA as a substrate and can form different dihydroceramide spices profiles (Riebeling et al., 2003). In final step of the *de novo* pathway, dihydroceramide is desaturated by dihydroceramide desaturase (DEGS) to form ceramide using molecular oxygen to introduce a hydroxyl group in the C-4 position of dihydroceramide, initiating a NADPH-aided dehydration reaction forming a double bond in the C4-C5 position (Kraveka et al., 2007). Ceramide can be further hydrolyzed to form sphingosine by ceramidases (CDase) (Figure 1B). Sphingosine is then phosphorylated by sphingosine kinase 1 and 2 (SK1/2) to generate sphingosine-1-phosphate (S1P) (Adams et al., 2020), a pro-survival signaling molecule that acts through 5 G-couple protein receptors (S1PR 1–5) (Cartier and Hla, 2019). S1P is quickly metabolized by S1P lyase to form hexadecenal and phosphoethanolamine (Zamora-Pineda et al., 2016).

Synthesis of more complex sphingolipids occurs in the Golgi, where glycosylation of ceramide yields complex glycosphingolipids, and phosphate or phosphocholine attachment yields ceramide-1-phosphate (C1P) (Sugiura et al., 2002). The addition of phosphocholine moiety onto the primary hydroxy group of ceramides by sphingomyelin synthase (SMS) forms sphingomyelin (SM) (Figure 1C). SM can also be recycled through the sphingomyelinase enzyme family, which hydrolyzes sphingomyelin’s phosphocholine headgroups to produce ceramide and free phosphocholine (D’Angelo et al., 2018). In mammals, sphingomyelinases can be separated into three main categories based on their optimal pH: acid sphingomyelinase (asmase), alkaline sphingomyelinase (aksmase), and neutral sphingomyelinase (nsmase). Asmase mostly resides in the lysosome (Beckmann et al., 2019) while nsmase is predominantly located on the plasma membrane (Schoenauer et al., 2019). In contrast, aksmase is only expressed in the intestine and liver and aids in the digestion of dietary sphingomyelin (Duan, 2006). Glycosphingolipids are distributed throughout subcellular compartments such as lysosomes and late endosomes where the acidic environment causes the degradation of the oligosaccharides, leading to the formation of ceramide (Kolter and Sandhoff, 2005). Acid ceramidase (ACDases) in the lysosome hydrolyzes ceramide into sphingosine and free fatty acid which leave the lysosome and are recycled back to ceramide (Figure 1D). This step is necessary due to ceramides’ inability to leave the lysosome (Chatelut et al., 1998). This ceramide recycling pathway is known as the salvage pathway and is estimated to be responsible for over half of all sphingolipid biosynthesis (Tettamanti et al., 2003).

### Sphingolipids and Aging

There is no doubt that SL metabolism plays an integral role in the regulation of almost every biological and physiological processes. Over the past 30 years, SL research has advanced significantly, illuminating and solidifying the importance of SL as a bioactive lipid species that regulates a vast number of biological functions. However, the structural diversity and the complexity of SL metabolism regulation still require a significant amount of research from multiple fields to comprehend the interconnected signaling pathways in addition to the function of each lipid species in a variety of pathological and physiological context. In the following section, we will provide a comprehensive overview of the link between SLs and aging with a particular focus on the major contributions of SLs to the hallmarks of aging and age-related diseases.

## Deregulated Nutrient Sensing

As nutrients are required for all biological processes, pathways that govern nutrient sensing are well characterized across many model organisms (Chantranupong et al., 2015). Deregulation of nutrient sensing is also arguably one of the most well studied mechanisms of aging and age-related diseases. Research over the past decade has discovered three major nutrient sensing pathways that play pivotal roles in energy homeostasis. These include AMP kinase (AMPK)/mammalian target of rapamycin (mTOR) and insulin/insulin-like growth factor 1 (IGF-1) signaling (IIS) (Templeman and Murphy, 2018). Recent studies have also linked SL metabolism to each of the these major nutrient sensing pathways and aging (Green et al., 2017).

AMPK signaling is a key component of metabolic energy sensing due to its ability to directly bind to adenine nucletodeis (Crozet et al., 2014). Changes in cellular energy (as measured by changes in the ratio of ATP/ADP and ATP/AMP) activates AMPK, signaling a cascade of downstream signaling pathways including lipid metabolism (Ahmadian et al., 2011), mitochondrial homeostasis (Toyama et al., 2016), glycolysis (Wu et al., 2013) and mTOR signaling (Inoki et al., 2003). AMPK activities have been shown to decrease with the increase of age (Reznick et al., 2007; Ljubicic and Hood, 2009), although the exact mechanisms for the decrease is unknown. Experimental activation of AMPK has been shown to increase lifespan in lower model organisms such as *C. elegans* (Apfeld et al., 2004) and *Drosophila* (Funakoshi et al., 2011). Interestingly, a 2014 study showed that when type 2 diabetics were treated with metformin, a AMPK activator, a 15% mean increase in lifespan was exhibited with matched non-diabetics (Bannister et al., 2014). Currently, the AMPK downstream effector mTOR has allured the attention of researchers worldwide, although there is evidence that suggests possible interactions between AMPK and sphingolipids. One study showed that treatment with myriocin (SPT inhibitor) in yeast extended the lifespan partly through the modulation of the AMPK pathways and protein kinase C downregulation (Liu et al., 2013a). While the S1P agonist drug FTY720 can activate protein phosphatase 2A (PP2A), leading to the dephosphorylation of AMPK at Thr172 and ultimately inducing cell death in multiple myeloma cells (Zhong et al., 2020).

mTOR signaling plays a central role in the sensing of nutrients, including insulin, amino acids and lipids (Um et al., 2004; Tokunaga et al., 2004). Disrupting S6K1, a mediator of mTOR signaling recapitulates metabolic profiles of caloric restriction (Choudhury, 2009), which is one of the most reliable ways of inducing prolonged lifespan in mammals (Madeo et al., 2019). Interestingly, age-related ceramide accumulation, more specifically, C16:0 has been shown to downregulate S6K1 in aging skeletal muscle (Rivas et al., 2012). Furthermore, recent transcriptomic study revealed that prolonged treatment with C16 ceramide-enriched lipoproteins downregulated several downstream phosphorylated intermediates of mTOR signaling pathway (Hammad, 2020). Moreover, cancer drugs such as doxorubicin, methotrexate and celecoxib increase C16 ceramide levels while reducing cell proliferation (Inoki et al., 2003; Denard et al., 2012; Fekry et al., 2016; Maeng et al., 2017). Overexpression of mammalian ceramide synthase CerS6 in human breast cancer cell line MCF-7 also results in accumulation of C16, leading to deceased cell proliferation due to inhibition of mTOR signaling through the reduction of phosphorylation of AKT, S6K and ERK. Similar inhibition was observed through C16 ceramide treatment as well (Kim et al., 2018). While C16 ceramide confers a negative regulation on mTOR, sphingosine-1-phosphate (S1P) exhibits a positive regulation by increasing mTOR signaling (Kim et al., 2018). This positive regulation has been shown in diverse cell types from primary fibroblasts to cancer cells. S1P interacts with the E3 ubiquitin ligase protein associated with Myc (PAM), which mediates GDP/GTP exchange of Ras homolog enriched in brain (RHEB), and directly activates mTOR signaling independent of ERL, AKT and P13 kinase (Maeurer et al., 2009). This differential regulation of opposing pathways between ceramide and S1P is known as the “SL Rheostat” coined in 1996 after several seminal discoveries demonstrating ceramide’s ability to induce cell growth arrest and apoptosis, while S1P was necessary for optimal cell proliferation and growth as well as the suppression of ceramide mediated growth arrest and apoptosis (Newton et al., 2015).

The insulin/insulin like growth factor signaling pathway (IIS) is perhaps the most promising metabolic pathways mediated by SLs. The IIS pathway is a highly complex metabolic regulatory system which is implicated in aging throughout multiple model organisms from nematodes to vertebrates (Khan et al., 2019). Studies over the last decade have demonstrated the ability of IIS to reconstitute the energy metabolic profile to benefit the short-term physiological needs by forgoing long-term organismal longevity and maintenance (Jęśko et al., 2019). In long lived *C. elegans*, defense mechanisms against stress conditions such as heat shock, oxidative stress and heavy metals are upregulated through the mediation of the IIS pathway (Lithgow and Walker, 2002) and this finding has been further corroborated in flies (Vermeulen and Loeschcke, 2007). Single nucleotide polymorphism studies in humans have suggested a link between the IIS signaling pathway and the determination of human longevity (Deelen et al., 2013), while genetic analysis of human female centenarians has also exhibited an over representation of mutations in insulin like growth factor I (IGF-I) receptor (IGF-IR) associated with reduced IGF-IR activity and increased serum IGF-I (Suh et al., 2008). Interestingly, IGF-IR is localized to membrane microdomains called lipid rafts that are dense in SLs and cholesterols, suggesting that IGF-I signaling is sensitive to changes in SL metabolism (Hong et al., 2004). Moreover, S1P’s ability to regulate cell fate and modulate energy homeostasis is analogous to that of IIS signaling (Green et al., 2017). IGF-IR activation involves the upregulation of sphingosine kinase and S1P receptor (S1PR) signaling, and the inhibition of either sphingosine kinase activity or the S1PR1 and S1PR3 receptors abolished EGF receptor activation potentiated by IGF binding protein 3 (IGFBP-3) in MCF-10A breast epithelial cells (Martin et al., 2009). Evidence suggest that IGFBP-3 is not only able to regulate the availability of IGF, but also exerts direct influence over apoptotic signaling *via* C2 ceramide mediated apoptosis (Perks et al., 1999). Furthermore, S1P activation of IGF-IR is mediated through its downstream signaling pathway targeting PI3K and Akt (Pyne et al., 2016). Ceramide can also target PI3K and Akt, inhibiting pro-survival signaling through the dephosphorylation of Akt by protein phosphatase (CAPP-PP2A) (Czubowicz and Strosznajder, 2014). Interestingly, ceramide-1-phosphate (C1P) exerts the opposite effect, inhibiting DNA fragmentation, PARP cleavage and caspase stimulation (Gómez-Muñoz, 2005).

Current data suggests extensive involvement of SL metabolism in the regulation of nutrient sensing and aging. The balance between ceramide and sphingosine no doubt contributes to the modulation of the nutrient sensing pathway towards longevity or short-term gains. Although these nutrient sensing pathways have been targets in worms, flies and mice with great success, it is still a topic that requires extensive research. A more comprehensive understanding of SLs is needed before we are able to target these pathways to modulate longevity in humans.

## Cellular Senescence

Cellular senescence is a state of arrested cell proliferation that can be triggered by critical stressors such as DNA damage, telomere shortening, and chromatin disruption. Interestingly, senescence has been shown to be protective and beneficial in the early stages of an organism’s life span (Muñoz-Espín, 2013), contributing to tissue repair and the protection against oncogenic factors. However, in aged organisms, chronic accumulation of senescent cells contributes to the decline of organismal health and age-related diseases (Childs et al., 2014). Recent evidence suggests several contributing factors of age-related accumulation of senescent cells. These include age-associated increase in senescence-inducing stimuli leading to an overall increase in senescent cells (Van Deursen, 2014), age-associated decline of immune function (Solana et al., 2012), and decrease in apoptotic regulation due to age-associated decline in P53 stability (Feng et al., 2007). Senescent cells can also influence neighboring cells in a paracrine manner through a complex secretome of cytokines and other signaling molecules, increasing inflammation and immune cell activation (Franceschi and Campisi, 2014). This phenomenon is referred to as the senescent associated secretory phenotype (SASP) and is the primary culprit in senescent related dysfunctions (Birch and Gil, 2020).

Recent studies have also highlighted the importance of SLs and their role in senescence regulation (Trayssac et al., 2018). Obeid *et al.* first noticed a significant increase in ceramide in senescent cells. Furthermore, the addition of exogenous ceramide mirrored several key factors of cellular senescence, including the inhibition of DNA synthesis and cell growth, and dephosphorylation of Retinoblastoma protein (Rb) (Venable et al., 1995). This was further recapitulated in human IMR-90 fibroblasts where ceramide level was doubled when comparing senescent to young fibroblast (Meacci et al., 1996). A link between aging tissue and N-SMase activity has also been reported (Jensen et al., 2005) along with a correlation between N-SMase activity and cellular senescence (Venable et al., 1995). However, ceramide may not be the only contributing factor to the induction of senescence. Acid ceramidase (ASAH1) which catalyzes ceramide into fatty acid and sphingosine has also been shown to be highly upregulated in senescent cells, and silencing ASAH1 in pre-senescent human fibroblasts decreased the expression levels of senescent associated proteins P53, P21, and P16 (Munk et al., 2021). Sphingosine-1-phosphate (S1P), whose role in promoting cellular proliferation and survival has been extensively studied (Proia and Hla, 2015), is generated through the phosphorylation of sphingosine by SK1/2 (Adams et al., 2020). The downregulation of SK1/SK2 or decreased level of cellular S1P has been shown to promote cellular senescence. Furthermore, S1P treatment and the inhibition of CerS reversed the acceleration in senescence, suggesting that the accelerated cellular senescence seen with SK1/2 inhibition was due to the increase in ceramide (Kim et al., 2019). Pharmacological inhibition of SK1/2 induced growth arrest in prostate cancer cells through the modulation of the ceramide synthesis pathway, which enhanced the expression of p53 and p21 (McNaughton et al., 2016). Although the exact mechanisms by which S1P regulates cellular senescence have not been established, evidence suggests that S1P may be an important regulator due to its ability to regulate cellular senescence through the binding of its target receptors. S1P has multiple binding targets, including five G-protein coupled receptors (S1PR1-5), which are responsible for a multitude of cellular functions (Cartier and Hla, 2019). S1PR2 activation induced senescence in endothelial cells that was reversed through S1PR2 shRNA (Lu et al., 2012). Interestingly, S1P can also bind to human telomeres reverse transcriptase (hTERT), mirroring hTERT phosphorylation and stabilizing hTERT and regulating cellular senescence caused by telomere damage (Panneer Selvam et al., 2015). While there is strong evidence highlighting the role of SLs in cellular senescence and aging, the precise mechanisms have yet to be elucidated. However, it would not be overreaching to speculate that the highly regulated intracellular balancing between the pro-survival and pro-apoptotic SLs contributes to the regulation of cellular senescence, and that this complex network comprises of key mediators that interconnect every aspect of cellular function.

## Loss of Proteostasis

Current understanding of protein homeostasis (proteostasis) and aging is supported by a magnitude of evidence suggesting that a properly functioning proteostasis network is associated with healthy aging, and many molecular interventions have aimed at diminishing the proteotoxic stress of an organism to increase its lifespan (Kaushik and Cuervo, 2015). The most well characterized networks that are responsible for proteostasis are the unfolded protein response (UPR). UPR is compartmentalized into UPR in the ER (UPR^ER^), mitochondrial UPR (UPR^mt^) and cytosolic UPR (UPR^cyt^) with each compartment possessing a unique framework of genes and proteins differentially regulated during times of stress (Kim et al., 2016). The UPR is responsible for detecting misfolded proteins and restoring it to its proper stable conformation (Kim et al., 2013) or utilizing a proteolytic network that drives the cell towards an autophagic cell fate and eliminates the dysfunctional cell if stress becomes chronic (Pohl and Dikic, 2019). Why our proteostasis network deteriorates as we age is still mostly unknown, however, evidence suggests that upon aging, the chaperone proteins responsible for maintaining proteostasis gradually amass oxidative damage, which lowers their efficiency at reenforcing proper protein refolding (Nuss et al., 2008). Inducibility of the UPR also declines with age in the UPR^ER^ as well as UPR^mt^ (Taylor, 2016). Furthermore, overall levels of chaperone proteins have also been shown to decrease with age, resulting in reduced proteostasis regulation and increased proteotoxicity (Taylor and Dillin, 2013).

The classical activators of UPR^ER^ are unfolded protein peptides which are recognized by three protein sensors IRE1a, PERK and ATF6a, initiating three separate signaling cascades of the UPR^ER^. However, recent evidence suggests that lipids can also regulate and activate the UPR^ER^. Perturbation of ER membrane lipid composition has been shown to activate UPR^ER^. Furthermore, IRE1a and PERK mutants with their luminal peptide sensing domain removed were able to activate UPR^ER^ in mammalian cells, suggesting the presence of a transmembrane lipid sensing domain (Volmer et al., 2013). Halbleib et al*.* also showed the presence of an amphipathic helix within the transmembrane domain of IRE1. The helices from two IRE1 proteins create transient dimerization configurations, which are stabilized during times of stress due to membrane compression, and upregulate UPR^ER^ activity (Halbleib et al., 2017).

The link between lipids and UPR has not been focused on SLs, however, the role of sphingolipids in membrane biology and their ability to manipulate membrane properties have caused an emergence of data. Tam et al. first showed an increase in dihydroceramide and dihydrosphingosine during activation of UPR^ER^. Furthermore, they were able to show that exogenous dihydroceramide and dihydrosphingosine were able to quickly enter the cell, upregulate ATF6, and induce nuclear localization in an array of mammalian cell lines (Tam et al., 2018). During the absence of stress, ATF6 is associated with BiP to inhibit its translocation to the nucleus, however, during times of stress, BiP disassociates from ATF6, allowing for translocation to the nucleus which initiates the UPR^ER^ signaling cascade (Susan, 2005). Interestingly, the activation of UPR^ER^ through dihydroceramide and dihydrosphingosine did not decrease the association between BiP and ATF6, suggesting that ATF6 exhibits a unique lipid sensing domain in addition to its protein peptide sensing domain that is able to upregulate UPR^ER^ (Tam et al., 2018).

The ER is essential to the maintenance of cellular proteostasis through its ability to activate the UPR and to reduce the protein aggregation burden *via* proteolytic degradation, translational regulation, proteasomal degradation, and autophagy (Hetz, 2012). For example, valosine containing protein (VCP), a ubiquitously expressed AAA + atpase removes stalled protein from ER membranes and targets it for proteasomal degradation (Bodnar and Rapoport, 2017). Mutations in VCP can lead to a number of developmental defects including inclusion body myopathy, Paget’s disease of the bone, amyotrophic lateral sclerosis and frontotemporal dementia or more commonly known as IBMPFD (Weihl et al., 2009). Interestingly, a lipid enriched diet (LED) was able to rescue the lethal phenotype of homozygous *VCP* ^*R155H*^ mutant mice as well as to improve disease progression. Furthermore, *VCP* ^*R155H*^ VCP R155H mutant mice observed increased levels of ceramide, which was decreased to a level closely resembling wild type when put on LED (Llewellyn et al., 2014). SLs has been linked to the disaggregation of polyglutamine (poly-Q) aggregates in *C. elegans* through the modulation of UPR^mt^ and UPR^cyt^. Reduced expression of the mitochondrial chaperone was able to induce the cytosolic stress response (UPR^cyt^) and to enhance poly-Q aggregate clearance in *C. elegans* expressing poly-Q35 repeats. Furthermore, microarray and lipidomic analyses determined that this unique stress response was regulated through lipid metabolism, specifically ceramide and sphingosine (Kim et al., 2016). This regulatory role of ceramide and sphingosine in organismal stress response and longevity is further supported by the NFYB-1-SPP-8 axis where perturbation of SL metabolism was able to modulate longevity through alteration of UPR^ER^ and UPR^mt^ (Tharyan et al., 2020).

## Sphingolipids and Other Hallmarks of Aging

The link between SLs and aging has become undeniable and the role of SLs in several aspects of aging hallmarks has been extensively studied, however, there are levels of complexity and regulations in other aspects that still require elucidating. Evidence have suggested that very long chain ceramide accumulates during the normal aging process (Cutler et al., 2004). Interestingly, long chain ceramides have been associated with mitochondrial dysfunction, leading to oxidative stress and cell death (Law et al., 2018). A study of mice comparing cardiac mitochondria in aged versus young animals showed that age associated accumulation of mitochondrial ceramide led to a declined mitochondrial function. This decline was reversed by treatment with (R)-α-lipoic acid (LA), a potent anti-oxidant/anti-inflammatory agent which reduced ceramide levels of aged mitochondria to a level comparable with young mitochondria (Monette et al., 2011). Furthermore, it was recently discovered that mitochondrial dysfunction as a result of age-associated ceramide accumulation is induced partly through the inhibition of PKA, leading to the activation of mitochondrial fission factor, dynamin-related protein 1 (Drp1), and ultimately mitophagy (Vaena et al., 2021).

DNA is subjected to damages from a variety of sources daily, which include exogenous factors such as ultraviolet radiation (UV), ionizing radiation (IR), a variety of chemical agents, toxins, and environmental stress, as well as endogenous factors consist of DNA replication error, spontaneous deamination and oxidative DNA damage. In response to the damage, cells utilize the DNA Damage Response (DDR) to mend the damages, thereby preventing prolonged stress and ultimately, cell death. Prolonged DNA damage can lead to genomic instability due to breaks in chromosomal DNA, unwanted mutations and cell death. Genomic instability has been well characterized for its role in cancer development, but its role in aging is still an ongoing research topic. However, there are strong evidence corroborating the notion that genomic instability is a strong driver of aging and age-related disease (Coppede and Migliore, 2012). The link between SLs and DDR was first shown in the early 1990s when increased sphingomyelinase activity and increased ceramide levels were observed in response to DNA damaging agents. The increase in ceramide was able to initiate cell cycle arrest through interactions with other DDR elements (Carroll et al., 2017). DNA damage was further shown to elicit a ceramide accumulation response that was P53 dependent (Dbaibo et al., 1998). This accumulation in ceramide was shown to be partially responsible for promoting cell cycle arrest in response to DNA damage (Phillips et al., 2007). P53 accumulation during DNA damage down regulates SK1, reducing the level of S1P in response to DNA damage. This occurs concomitantly with ceramide accumulation, possibly due to the pro-apoptotic response associated with chronic DNA damage (Taha et al., 2004). Current research has identified several SL metabolites and enzymes that regulate cellular DDR with the potential of offering new therapeutic interventions against DNA damage caused by cancer agents. However, the link between SLs and DDR when pertaining to the aging process requires elucidation.

The role of epigenetic regulation in aging is a topic that has been drawing the attention of researchers since the 1960s (Berdyshev et al., 1967). Chronological age was an imprecise determinant of the aging process, and so the search for biomarkers that could determine biological age began (Baker and Sprott, 1988). The discovery of DNA methylation and how specific CpG methylation sites change throughout aging (Hernandez et al., 2011) has led to the advent of the “Epigenetic Clock”. By utilizing DNA material from blood or tissue, the epigenetic clock estimates the biological age of the specimen through the use of complex mathematic algorithms and advance DNA methylation arrays (Horvath and Raj, 2018). The relationship between the epigenetic regulation of sphingolipids and its impact on the aging process is a topic that has not been well studied, however, recent studies on this topic provides new and exciting insights. Hait *et al.* showed that nuclear S1P can target histone deacetylase HDAC1 and HDAC2, possibly regulating chromatin state in response to environmental stimulus (Hait et al., 2009). This interaction was further corroborated in mice where the increase in S1P level diminished nuclear HDAC activity, resulting in increase in fatty acid metabolism genes and decrease in inflammation genes (Nguyen-Tran et al., 2014).

Stem cell exhaustion has also been linked to the process of aging although the research density of SLs and their role in stem cell exhaustion is quite low. Non-canonical Wnt signaling is required for the regulation of stem cells essential for development as well as disease (Sugimura and Li, 2010). In the brain, non-canonical Wnt signaling induces stemness of neuronal stem cells. Very long chain ceramide has been shown to regulate the nonconical Wnt signaling pathway through glycogen synthase kinase 3 (GSK3) (Kong et al., 2015). Incubation with long chain ceramide was able to promote differentiation in human embryonic stem cells (hESC) and induced pluripotent stem cell (iPSC) derived neuroprogenitors (He et al., 2014). Interestingly, S1P has also been shown to mediate hESC self-renewal as well as mouse embryonic stem cell differentiation (Rodgers et al., 2009).

As the lipid landscape changes throughout the aging process, altered intercellular communication occurs as a product of several aberrant physiological processes involving SLs. As mentioned above, lipid rafts are micro domains primarily constructed of SLs and cholesterol. They serve as signaling hubs for a diverse range of cellular processes including proliferation, migration and survival (Mollinedo and Gajate, 2015). However, age-related change in SL levels disrupts the microdomain, leading to altered cellular signaling, resulting in neuropathologies (Mesa-Herrera et al., 2019). Age related increase in senescent cells contributes to altered intercellular communication through the senescence-associated secretory phenotype (SASP). Senescent cells can trigger neighboring cells to enter senescence through gap junction mediated cell to cell contact, propagating senescence to nearby cells (Nelson et al., 2012). The SASP results in an imbalance of cytokine expression and altering cellular communication towards a pro-inflammation state. This phenomenon is known as “inflammaging” and is described in extensive detail by Rea et al (2018).

## Sphingolipids and Age-Related Diseases

### Neurodegenerative Diseases

**FIGURE 2:**
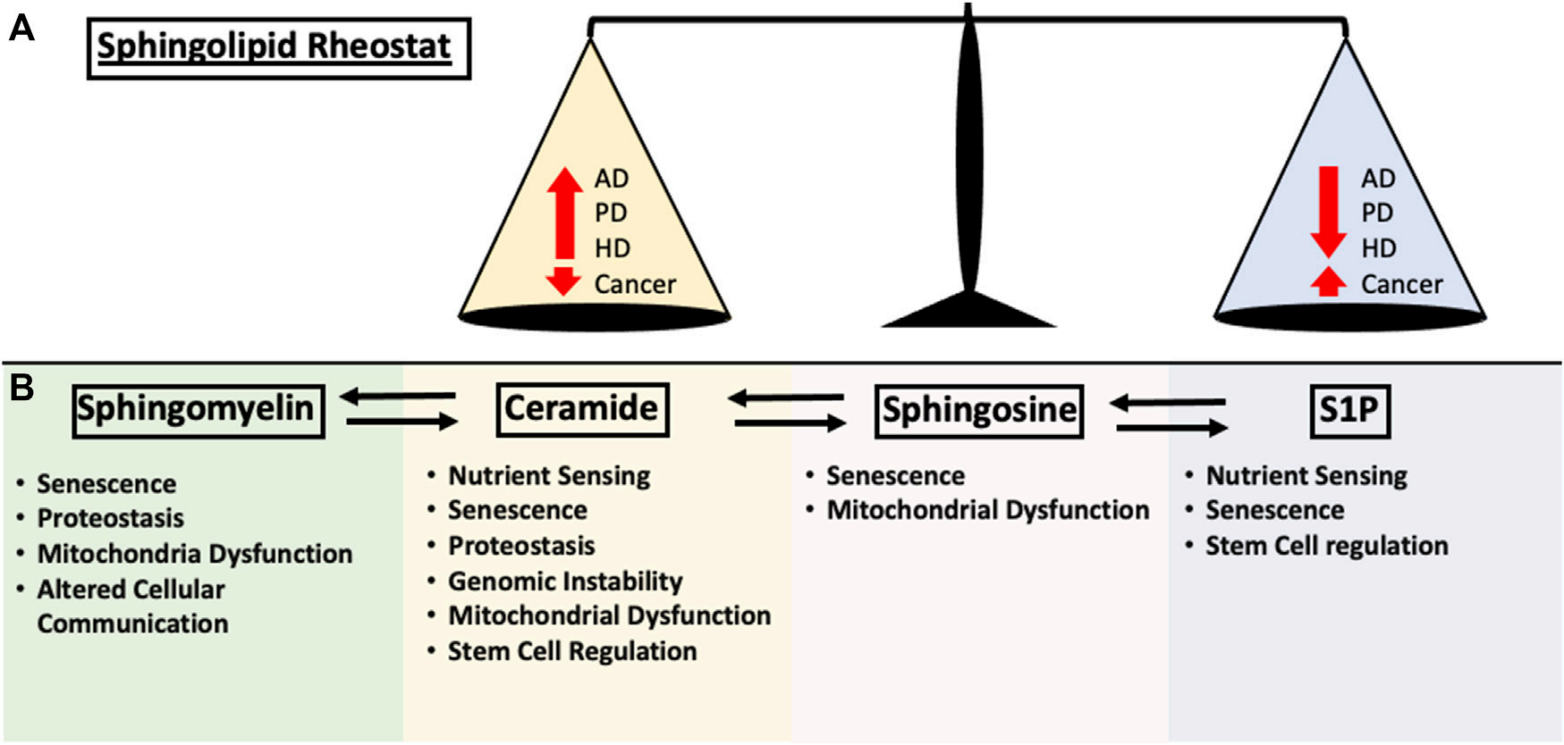
Summary of the impact each sphingolipid species have on the hallmarks of aging discussed in this review. **(A)** Early sphingolipid research uncovered the differential regulation of cell fate through the modulation of signaling pathways. Ceramide upregulation resulted in the activation of apoptosis and cell growth arrest while S1P is required for proper cellular growth. This is now known as the “Sphingolipid Rheostat” and is one of the fundamental concepts of sphingolipid biology. In age-related disease such as Alzheimer’s Disease (AD), Huntington’s Disease (HD), Parkinson’s Disease (PD) and Cancer, the alterations in sphingolipid metabolism follows the rheostat. Changes in either pro-apoptotic or pro-survival signaling pathway is negatively correlated in the other. **(B)** Contributions of the major sphingolipid metabolites to the hallmarks of aging as mentioned in this review.

The complex proteostasis network responsible for managing misfolded proteins is subjected to extensive regulations, however, as we age, our proteostasis network begins to deteriorate due to the accumulation of oxidative damage and overall decrease in chaperone levels, causing increased protein aggregation stress on the cell and leading to disease states (Martínez et al., 2017). A deteriorating UPR could lead to the accumulation of neurotoxic aggregates associated with neurodegenerative diseases. Protein aggregation is considered a defining characteristic in multiple neurodegenerative disease which includes Amyloid Beta (Aβ) and Hyperphosphorylated tau (Tau) in Alzheimer’s Disease (AD), Alpha-synuclein (α-syn) in Parkinson’s Disease (PD) and poly-glutamine (poly-Q) in Huntington’s Disease (HD). Although the protein aggregate associated with each of the disease is different, aging is a common risk factor that is shared among them (Kurtishi et al., 2019). When the protein aggregate burden begins to outpace the proteostasis network, neurotoxic proteins can accumulate, leading to disease-associated protein aggregation (Tanaka and Matsuda, 2014). In the past decade, research has suggested regulatory roles of SL in neuronal cell growth, differentiation, survival and apoptosis (Hirabayashi and Furuya, 2008). Due to the SL-rich composition of the brain as well as the complex involvement of SL in brain physiology, it comes as no surprise that aberrant SL metabolism is linked to neurodegenerative diseases (Haughey, 2010). Due to the interconnectivity of the SL metabolism, changes in a single node of the metabolic pathway could lead to dramatic changes in subsequent metabolic intermediates. The sphingolipid rheostat was established to encapsulate the differential regulation of cellular fitness (Cuvillier et al., 1996). Interestingly, age-related disease exhibits a similar theme of SL alterations, displaying an inverse balance between pro-survival and pro-death SL signaling (Figure 2A). The following section will summarize several key involvements of SLs in AD, PD and HD.

#### Alzheimer’s Disease

In AD, SL metabolism has been shown to be perturbed in patients even before the manifestation of clinical symptoms, and tipping the balance of SLs to favor ceramide contributes to the overall disease progression of AD (Varma et al., 2018). SLs, along with cholesterol, form a lipid rich microdomain called lipid rafts which house many signaling molecules and have been associated with aging and neurodegeneration (Ohno-Iwashita et al., 2010). Interestingly, amyloid precursor protein (APP), along with several key enzymes responsible for the cleavage of APP, including BACE-1, have all been shown to co-localize to lipid rafts (Hicks et al., 2012). The disruption of lipid raft through cholesterol depletion showed an inhibition in the formation of Aβ (Simons et al., 1998). SL deficiency has also been shown to alter APP cleavage, possibly through the alteration of lipid raft structure interfering with function (Sawamura et al., 2004). However, ceramide was shown to increase beta APP cleavage through the stabilization of BACE-1, leading to increased Aβ production (Puglielli et al., 2003). The main genetic determinant of AD is the polymorphic alleles of ApoE, a cholesterol carrier whose implication in AD has been extensively studied (Liu et al., 2013b). Kurano *et al.* have shown that ApoE accelerates the clearance of circulating S1P and ApoM (Kurano et al., 2015), which further emphasize the importance of SLs in AD and aging.

Ceramide accumulation is associated with early stages of AD while an overall decrease in ceramide levels is observed in late stage AD (Katsel et al., 2007). An increase in ceramide levels in early stages of AD sensitizes neuronal cells to pro-death regulation (Jana1 et al., 2009). Furthermore, this increase in ceramide is a direct result of CerS activation, which increases *de novo* ceramide synthesis, particularly of the C22:0 and C24:0 species (Katsel et al., 2007). Interestingly, Aβ has also been shown to mediate the increase in SPT activity, resulting in increased *de novo* synthesis of ceramide. The inhibition of SPT in mice resulted in decreased SPT and ceramide levels along with a decline in Aβ and hyperphosphorylated tau (Hirosha Geekiyanagea et al., 2013). Although the *de novo* pathway is responsible for the early increase in ceramide levels, the main source of ceramide in AD originates from the hydrolysis of sphingomyelin as a result of Aβ (). In postmortem AD patients, analysis of senile plaques showed increased levels of ceramide saturation and nsmase/asmase (Panchal, 2014). Aβ induces neuronal apoptosis through the upregulation of ceramide by nsmase and asmase (Malaplate-Armand, 2006). Although the implications of the accelerated S1P clearance in AD requires elucidation, one can speculate that the decreasing plasma levels of S1P over the course of aging could possibly tip the balance of SL regulation towards pro-death. This, along with the Aβ mediated increase in ceramide levels could result in a cascade of ceramide mediated neuronal cell death and exacerbate the AD phenotype.

#### Parkinson’s Disease

One of the defining characteristics of PD is the presence of Lewy Bodies (LB) which are fibrillar aggregates mainly composed of α-syn (Wakabayashi et al., 2007). Deletion of the gene coding for α-syn (SNCA) has been linked to altered SL metabolism (Golovko, 2005) and dysfunction in SL metabolism has been associated with the presence of Lewy bodies (Rocha, 2015). Recent evidence suggests aberrant endolysosomal pathways play a key role in the development of PD. Due to the importance of membrane dynamics in proper lysosomal function, altered SLs may contribute to lysosomal dysfunction and the exacerbation of PD (Song et al., 2016). The autophagy-lysosome pathway is responsible for the removal of toxic protein aggregates such as α-syn. Impaired function could lead to the accumulation of neurotoxic aggregates, leading to PD state (Pan et al., 2008). Mutation in PLA2G6, a phospholipase gene associated with PD and PD-like disease onset resulted in neurotoxicity due to increased levels of ceramide *via* increased ceramide recycling. Reduction in ceramide through phenological or genetic manipulation resolved PLA2G6 neurotoxic phenotype in flies (Lin, 2018). This was further corroborated in PD associated VPS35 gene variant where improved fitness was achieved through ceramide manipulation (Lin, 2018). Gaucher Disease (GD) is a lysosome storage disease (LSD) caused by biallelic mutation in the GBA (acid beta-glucocerebrocidase) gene, which encodes a SL metabolism enzyme. Patients with a heterozygous recessive variants of GBA have significantly increased risk of PD (Sidransky et al., 2010). Furthermore, loss of function mutation in GBA inhibited the autophagy-lysosome pathway, resulting in increased α-syn levels (Du et al., 2015). Brain specimens taken from GD patients exhibited α-syn positive lewy bodies (Wong, 2004), while a loss of GBA in human pluripotent-stem-cell derived dopamine neurons led to neuronal death due to α-syn accumulation induced lysosome dysfunction (Mazzulli, 2011). The role of S1P has also been explored in PD. pharmacological inhibition of SK1 reduced survival of dopaminergic neurons in a PD mouse model while exogenous S1P exhibited neuroprotective effects through the inhibition of apoptosis (Pyszko and Strosznajder, 2014). Treatment with S1P antagonist FTY720 significantly reduced neuromotor defects and attenuated the decrease in neuronal cell fitness in several mouse PD models (Zhao, 2017).

#### Huntington’s Disease

HD is a genetic neurodegenerative disease that is autosomal dominant and presents with a wide range of physical, behavioral and cognitive defects (Vonsattel and DiFiglia, 1998). Similar to other neurodegenerative disease, alterations in SL metabolism can be detected early on in the HD pathology (Pardo et al., 2017). Recent studies have suggested a link between altered SL metabolism and HD pathology. S1P levels in HD mouse models exhibited upregulation of S1P lyase (SGPL1) as well as a downregulation in SphK1. This was further corroborated in human striatal and cortical specimens from human HD patients (Pardo, 2017). Treatment with K6PC-5 (SphK1 activator) in HD mouse model resulted in increased S1P levels and improved intestinal cell integrity and prevented body weight loss (Pardo, 2020). K6PC-5 treatment also further prevented motor defects in R6/2 HD mice model as well as reduced mutant huntingtin aggregates. This was accomplished through the activation of pro-survival signaling and increased autophagy (Di Pardo, 2019). S1P manipulation through drug targeting has shown positive results in improving HD pathology. FTY720 treatment of R6/2 mice showed improved lifespan and motor function along with reduced brain atrophy. Furthermore, FTY720 treatment reduced the levels of cytotoxic signaling molecules that would otherwise lead to neurotoxic astrocyte activation (Pardo et al., 2014; Miguez et al., 2015). A-971432 (S1PR5 Agonist) treatment of R6/2 HD mice significantly improved lifespan. Additionally, when A-971432 treatment was administered early, prior to the onset of disease symptoms, it protected the mice from HD associated progressive neuromotor defects (Pardo et al., 2018). Although research in the relationship between SLs and HD is scattered, there is promising evidence suggesting that the targeting of the S1P axis will improve HD pathology and disease management.

### Cancer

One of the biggest risk factors for cancer is old age (Niccoli and Partridge, 2012). It is no coincidence that the hallmarks of aging strikingly resembles the hallmarks of cancer (Hanahan and Weinberg, 2011). Aging confers genomic instability due to the accumulation of DNA mutations which ultimately grants a gain-of-function mutation, allowing the cell to proliferate uncontrollably, migrate and survive the immune system (Hanahan and Weinberg, 2011). While some cells proliferate due to gain-of-function mutations, others enter a senescent state, preventing them from propagating their DNA damage (Coppé et al., 2010). Along with mutation accumulation, telomere shortening due to continued replication throughout aging induces senescence as well (Bernadotte et al., 2016). It comes as no surprise that senescent cell numbers also increase with age, however, increase cellular senescence is a potent driver of hyperplasia (Robert et al., 2020).

Recent evidence have suggested that the modulation of SLs also contributes significantly to cancer pathology (Sheng, 2018). S1P has been shown to exert pro-survival effects on cancer cells, promoting proliferation, migration, transformation and inhibiting ceramide induced apoptosis (Cuvillier et al., 1996), while ceramide confers sensitivity to chemotherapeutic drugs, senescence, cancer cell apoptosis and growth arrest (Morad and Cabot, 2013). Recent studies have solidified the role of Sphingosine kinase 1 (SK1) in cancer (Heffernan-Striud and Obeid, 2013). This is best documented in human colon cancer where 89% of cases exhibit upregulation in SK1 ^6^. Sk1 knockout mice exhibited inhibitory effects in colon tumor development as well as increased apoptotic colon cancer cells (Kawamori, 2009). Along with the increase in S1P through SK1, S1P lyase downregulation in colon cancer has also been documented, further increasing pro-survival S1P signaling (Oskouian, 2006). Many of the current chemotherapeutic drugs have been shown to increase ceramide generation through several key synthesis pathways. Increased cellular S1P through the siRNA inhibition of S1P phosphatase one in MCF-7 cells conferred resistance to chemotherapeutic agent daunorubicin (Johnson, 2003). Other chemotherapeutic agents such as Gemcitabine (Chalfant, 2002) and doxorubicin (Lépine et al., 2011) increased ceramide levels through the *de novo* pathway which can be partially inhibited with treatment of myriocin, an inhibitor of SPT. Daunorubicin upregulates ceramide synthase activity, resulting in increased *de novo* ceramide synthesis (Bose, 1995). Cytosine arabinoside increases ceramide levels through the activation of N-SMase by generating reactive oxygen species (Bezombes, 2001). The reduced level of ceramide in cancer cells drives the cells towards a pro-survival phenotype. This can be further validated in the upregulation of acid ceramidase (acdase) enzyme in several cancer types, which is responsible for the degradation of ceramide into sphingosine (Mahdy, 2009; Bizzozero, 2014). Inhibition of acdase combined with chemotherapeutic drugs has shown promise in inducing cancer cell death (Realini, 2016).

### Therapy Targeting Sphingolipids

The multilayered regulatory synthesis pathway of sphingolipid metabolism provides many opportunities for pharmacological intervention. However, most sphingolipid therapies revolve around age-related disease, and little is known about its effects on aging. The forerunner of sphingolipid therapy is a S1PR modulating drug called FTY720, which is currently FDA-approved for the treatment of relapsing remitting forms of multiple sclerosis (MS) (Brinkmann, 2002). FTY720 has also been heavily researched as an anti-cancer therapeutic in a multitude of organ systems including leukemia (Neviani, 2007), glioblastoma (Estrada-Bernal et al., 2012), breast cancer (Azuma, 2002), non-small cell lung cancer (Booth et al., 2019), pancreatic cancer (Lankadasari, 2018) and mesothelioma (Szymiczek, 2017). Targeting the SphK-S1P-S1PR pathway has been a promising anti-cancer strategy that has given rise to many other candidates. For example, SK1-I and PF-543, two potent SphK1 inhibitors, suppress the growth of glioblastoma (Kapitonov, 2010) and colorectal cancer cells (Ju et al., 2016) in xerograph mouse model, respectively. Other drugs targeting S1PR1/3 (VPC03090) (Kennedy, 2011) and S1PR2 (AB1) (Li et al., 2015) are also under further evaluation to be possible future anti-cancer drugs. Currently, there are five drugs that reproducibly extend lifespan in mice: rapamycin, acarbose, nordihydroguaiartic acid, 17-alpha-oestradiol and aspirin (Nadon et al., 2017). Of the five, rapamycin presents an interesting topic for future sphingolipid research due to mTORC being the target for its mechanism of action (Schreiber, 2019). Identifying the change in sphingolipid landscape under the influence of rapamycin induced lifespan extension could provide a more coherent view of how sphingolipids regulate lifespan, as well as provide insight for the development of new pharmacological interventions. One interesting field of research that is currently ongoing is the use of sphingolipids as a biomarker for aging. Serum sphingolipids have been proposed to serve as biomarkers of obesity (Mu, 2013) and AD (Mielke and Haughey, 2012). While serum C16 shows promise as a biomarker for gait speed, a robust measurement for physical functional decline (Wennberg, 2018). The usefulness of sphingolipid as a biomarker is further supported by lipidomic data from centenarians (Montoliu, 2014), solidifying the need for further exploration to identify the relationship between sphingolipid metabolism and the aging process.

## Conclusion

The impact of SLs can be observed in almost every field of aging research. In this review, we provided a complete summary of sphingolipids and their role in the hallmarks of aging (Figure 2B). However, the complexity in their diversity and regulation is reflected in the fragmented data in their roles in age regulation. Although recent evidence due suggest that targeting the SL metabolism pathway alters lifespan, the underlining molecular pathways need further elucidating. Furthermore, the biochemical and molecular mechanisms that govern the regulation of enzymes involved in the SL metabolism pathway, as well as the mechanistic action of the many species of SL intermediates requires further unraveling. Due to the interconnectedness of SLs, a comprehensive understanding of SL structure, subcellular localization and the regulation of its enzymes and pathway will be required for safe and effective modulation of the aging process, in addition to enabling the development of precise and effective therapeutic tools in the fight against age-related diseases. Although the hallmarks represent a foundation in which aging can be systematically defined, it is by no means a complete picture of the aging process. Science is still far from fully understanding the aging process, and the challenges of studying aging and longevity in humans have proven extremely difficult. In mice, therapeutic targets aimed at several hallmarks of aging can increase lifespan by 15–20%, however, this comes at a cost of decreased fitness and physiological defects. This cost for longevity would explain the absence of long-lived phenotypes in humans due to the importance of survival fitness. Ongoing research into centenarians has shed some light into the aging process, characterizing lipid profiles and genetic contributing factors of centenarians and their offspring compared to the average human. As scientists slowly unravel the intricacies of aging, the interconnected network of SL metabolism and its implications in aging is going to be an exciting and dynamic field for future research.

## Glossary

AD: Alzheimer’s Disease
AkSMase: Alkaline Sphingomyelinase
AMPK: AMP activated protein kinase
APP: Amyloid Precursor Protein
ASAH1: Acid Ceramidase
ASMase: Acid Sphingomyelinase
ATK: Protein Kinase B
ATP: Adenosine Triphosphate
Aβ: Amyloid Beta
C1P: Ceramide-1-Phosphate
CDase: Ceramidases
CerS: Ceramide Synthase
DAG: Diacylglycerol
DDR: DNA Damage Response
DEGS: Dihydroceramide Desaturase
ER: Endoplasmic Reticulum
FA: Fatty Acid
GL: Glycerolipids
GP: Glycerophospholipids
HD: Huntington’s Disease
hTERT: Human Telomerase Reverse Transcriptase
IGF: Insulin Growth Factor
IIS: Insulin/Insulin Like Growth Factor Signaling
LB: Lewy Bodies
LED: Lipid Enriched Diet
mTOR: Mammalian Target of Rapamycin
NSMase: Netural Sphingomyelinase
PD: Parkinson’s Disease
PK: polyketides
PKC: Protein Kinase C
PolyQ: Poly Glutamine
PR: Phenol Lipids
Rb: Retinoblastoma Protein
S1P: Sphingopsine-1-Phosphate
S1PR: Sphingosine-1-Phosphate Receptor
SaL: Saccharolipids
SASP: Senescent Associated Secretory Phenotype
SK1/2: Sphingosine Kinase
SL: Sphingolipids
SM: Sphingomyelin
SMS: Sphingomyelinase
SPT: Serine Palmitoyltransferase
ST: Sterol Lipids
UPR: Unfolded Protein Response
VCP: Valosine Containing Protein

## References

[B1] Adams D. R. Pyne S. Pyne N. J. (2020). Structure-function Analysis of Lipid Substrates and Inhibitors of Sphingosine Kinases. Cell Signal. 76, 109806. 10.1016/j.cellsig.2020.109806 33035646

[B2] Ahmadian M. Abbott M. J. Tang T. Hudak C. S. S. Kim Y. Bruss M. (2011). Desnutrin/ATGL Is Regulated by AMPK and Is Required for a Brown Adipose Phenotype. Cell Metab. 13, 739–748. 10.1016/j.cmet.2011.05.002 21641555 PMC3148136

[B3] Apfeld J. O'Connor G. McDonagh T. DiStefano P. S. Curtis R. (2004). The AMP-Activated Protein Kinase AAK-2 Links Energy Levels and Insulin-like Signals to Lifespan in *C. elegans* . Genes Dev. 18, 3004–3009. 10.1101/gad.1255404 15574588 PMC535911

[B4] Azuma H. (2002). Marked Prevention of Tumor Growth and Metastasis by a Novel Immunosuppressive Agent, FTY720, in Mouse Breast Cancer Models. Cancer Res. 62, 1410–1419. 11888913

[B5] Baker G. T. Sprott R. L. (1988). Biomarkers of Aging. Exp. Gerontol. 23, 223–239. 10.1016/0531-5565(88)90025-3 3058488

[B6] Bannister C. A. Holden S. E. Jenkins-Jones S. Morgan C. L. Halcox J. P. Schernthaner G. (2014). Can People with Type 2 Diabetes Live Longer Than Those without? A Comparison of Mortality in People Initiated with Metformin or Sulphonylurea Monotherapy and Matched, Non-diabetic Controls. Diabetes Obes. Metab. 16, 1165–1173. 10.1111/dom.12354 25041462

[B7] Beckmann N. Becker K. A. Kadow S. Schumacher F. Kramer M. Kühn C. (2019). Acid Sphingomyelinase Deficiency Ameliorates farber Disease. Ijms 20, 6253. 10.3390/ijms20246253 PMC6941101 31835809

[B8] Berdyshev G. D. Korotaev G. K. Boiarskikh G. V. Vaniushin B. F. (1967). [Nucleotide Composition of DNA and RNA from Somatic Tissues of Humpback and its Changes during Spawning]. Biokhimiia 32, 988–993. 5628601

[B9] Bernadotte A. Mikhelson V. M. Spivak I. M. (2016). Markers of Cellular Senescence. Telomere Shortening as a Marker of Cellular Senescence. Aging (Albany. NY) 8, 3–11. 10.18632/aging.100871 26805432 PMC4761709

[B10] Bezombes C. (2001). Oxidative Stress‐induced Activation of Lyn Recruits Sphingomyelinase and Is Requisite for its Stimulation by Ara‐C. FASEB J. 15, 1583–1585. 10.1096/fj.00-0787fje 11427493

[B11] Birch J. Gil J. (2020). Senescence and the SASP: Many Therapeutic Avenues. Genes Dev. 34, 1565–1576. 10.1101/gad.343129.120 33262144 PMC7706700

[B12] Bizzozero L. (2014). Acid Sphingomyelinase Determines Melanoma Progression and Metastatic Behaviour via the Microphtalmia-Associated Transcription Factor Signalling Pathway. Cell Death Differ. 21, 507–520. 10.1038/cdd.2013.173 24317198 PMC3950316

[B13] Bodnar N. Rapoport T. (2017). Toward an Understanding of the Cdc48/p97 ATPase. F1000Res 6, 1318–1410. 10.12688/f1000research.11683.1 28815021 PMC5543420

[B14] Booth L. Roberts J. L. Spiegel S. Poklepovic A. Dent P. (2019). Fingolimod Augments Pemetrexed Killing of Non-small Cell Lung Cancer and Overcomes Resistance to ERBB Inhibition. Cancer Biol. Ther. 20, 597–607. 10.1080/15384047.2018.1538616 30388910 PMC6605998

[B15] Bose R. (1995). Ceramide Synthase Mediates Daunorubicin-Induced Apoptosis: An Alternative Mechanism for Generating Death Signals. Cell 82, 405–414. 10.1016/0092-8674(95)90429-8 7634330

[B16] Brinkmann V. (2002). The Immune Modulator FTY720 Targets Sphingosine 1-phosphate Receptors. J. Biol. Chem. 277, 21453–21457. 10.1074/jbc.c200176200 11967257

[B17] Bustos V. Partridge L. (2017). Good Ol' Fat: Links between Lipid Signaling and Longevity. Trends Biochem. Sci. 42, 812–823. 10.1016/j.tibs.2017.07.001 28802547 PMC6231542

[B18] Carroll B. Donaldson C. Obeid L. (2017). Sphingolipids in the DNA Damge Response. Physiol. Behav. 176, 139–148. 28363838

[B19] Cartier A. Hla T. (2019). Sphingosine 1-phosphate: Lipid Signaling in Pathology and Therapy. Science 366. 10.1126/science.aar5551 PMC7661103 31624181

[B20] Chalfant C. E. (2002). De Novo ceramide Regulates the Alternative Splicing of Caspase 9 and Bcl-X in A549 Lung Adenocarcinoma Cells. Dependence on Protein Phosphatase-1. J. Biol. Chem. 277, 12587–12595. 10.1074/jbc.m112010200 11801602

[B21] Chantranupong L. Wolfson R. L. Sabatini D. M. (2015). Nutrient-Sensing Mechanisms across Evolution. Cell 161, 67–83. 10.1016/j.cell.2015.02.041 25815986 PMC4384161

[B22] Chatelut M. Leruth M. Harzer K. Dagan A. Marchesini S. Gatt S. (1998). Natural Ceramide Is Unable to Escape the Lysosome, in Contrast to a Fluorescent Analogue. FEBS Lett. 426, 102–106. 10.1016/s0014-5793(98)00325-1 9598987

[B23] Childs B. G. Baker D. J. Kirkland J. L. Campisi J. Deursen J. M. (2014). Senescence and Apoptosis: Dueling or Complementary Cell Fates? EMBO Rep. 15, 1139–1153. 10.15252/embr.201439245 25312810 PMC4253488

[B24] Choudhury A. I. (2009). Ribosomal Proteion S6 Kinase 1 Signaling Regulates Mammalian Life Span. Science 461, 140–144. 10.1126/science.1177221 PMC4954603 19797661

[B25] Coppé J.-P. Desprez P.-Y. Krtolica A. Campisi J. (2010). The Senescence-Associated Secretory Phenotype: The Dark Side of Tumor Suppression. Annu. Rev. Pathol. 8, 99–118. 10.1146/annurev-pathol-121808-102144 PMC4166495 20078217

[B26] Coppede F. Migliore L. (2012). DNA Repair in Premature Aging Disorders and Neurodegeneration. Curr. Aging Sci. 3, 3–19. 10.2174/1874609811003010003 20298165

[B27] Crozet P. Margalha L. Confraria A. Rodrigues A. r. Martinho C. u. Adamo M. (2014). Mechanisms of Regulation of SNF1/AMPK/SnRK1 Protein Kinases. Front. Plant Sci. 5, 190–217. 10.3389/fpls.2014.00190 24904600 PMC4033248

[B28] Cutler R. G. Kelly J. Storie K. Pedersen W. A. Tammara A. Hatanpaa K. (2004). Involvement of Oxidative Stress-Induced Abnormalities in Ceramide and Cholesterol Metabolism in Brain Aging and Alzheimer's Disease. Pnas 101, 2070–2075. 10.1073/pnas.0305799101 14970312 PMC357053

[B29] Cuvillier O. Pirianov G. Kleuser B. Vanek P. G. Coso O. A. Gutkind J. S. (1996). Suppression of Ceramide-Mediated Programmed Cell Death by Sphingosine-1-Phosphate. Nature 381, 800–803. 10.1038/381800a0 8657285

[B30] Czubowicz K. Strosznajder R. (2014). Ceramide in the Molecular Mechanisms of Neuronal Cell Death. The Role of Sphingosine-1-Phosphate. Mol. Neurobiol. 50, 26–37. 10.1007/s12035-013-8606-4 24420784 PMC4181317

[B31] D’Angelo G. Moorthi S. Luberto C. (2018). Role and Function of Sphingomyelin Biosynthesis in the Development of Cancer. Adv. Cancer Res. 140, 61–96. 30060817 10.1016/bs.acr.2018.04.009

[B32] Dbaibo G. S. Pushkareva M. Y. Rachid R. A. Alter N. Smyth M. J. Obeid L. M. (1998). P53-Dependent Ceramide Response to Genotoxic Stress. J. Clin. Invest. 102, 329–339. 10.1172/jci1180 9664074 PMC508891

[B33] Deelen J. Uh H.-W. Monajemi R. van Heemst D. Thijssen P. E. Böhringer S. (2013). Gene Set Analysis of GWAS Data for Human Longevity Highlights the Relevance of the insulin/IGF-1 Signaling and Telomere Maintenance Pathways. Age 35, 235–249. 10.1007/s11357-011-9340-3 22113349 PMC3543749

[B34] Denard B. Lee C. Ye J. (2012). Doxorubicin Blocks Proliferation of Cancer Cells through Proteolytic Activation of CREB3L1. Elife 1, 1–14. 10.7554/eLife.00090 PMC3524649 23256041

[B35] Di Pardo A. (2019). Stimulation of Sphingosine Kinase 1 (SPHK1) Is Beneficial in a Huntington’s Disease Pre-clinical Model. Front. Mol. Neurosci. 12, 1–11. 10.3389/fnmol.2019.00100 31068790 PMC6491579

[B36] Dinkins M. B. (2016). Neutral Sphingomyelinase-2 Deficiency Ameliorates Alzheimer’s Disease Pathology and Improves Cognition in the 5XFAD Mouse. J. Neurosci. 36, 8653–8667. 10.1523/jneurosci.1429-16.2016 27535912 PMC4987436

[B37] Du T.-T. Wang L. Duan C. L. Lu L. L. Zhang J. L. Gao G. (2015). GBA Deficiency Promotes SNCA/α-synuclein Accumulation through Autophagic Inhibition by Inactivated PPP_2_A. Autophagy 11, 1803–1820. 26378614 10.1080/15548627.2015.1086055 PMC4824589

[B38] Duan R. (2006). Alkaline Sphingomyelinase: An Old Enzyme with Novel Implications. Biochim. Biophys. Acta (BBA) - Mol. Cell Biol. Lipids 1761, 281–291. 10.1016/j.bbalip.2006.03.007 16631405

[B39] Estrada-Bernal A. Palanichamy K. Chaudhury A. R. Van Brocklyn J. R. (2012). Induction of Brain Tumor Stem Cell Apoptosis by FTY720: A Potential Therapeutic Agent for Glioblastoma. Neuro. Oncol. 14, 405–415. 10.1093/neuonc/nos005 22351749 PMC3309856

[B40] Fahy E. Subramaniam S. Murphy R. C. Nishijima M. Raetz C. R. H. Shimizu T. (2009). Update of the LIPID MAPS Comprehensive Classification System for Lipids. J. Lipid Res. 50, S9–S14. 10.1194/jlr.R800095-JLR200 19098281 PMC2674711

[B41] Fekry B. Esmaeilniakooshkghazi A. Krupenko S. A. Krupenko N. I. (2016). Ceramide Synthase 6 Is a Novel Target of Methotrexate Mediating its Antiproliferative Effect in a P53-dependent Manner. PLoS One 11, e0146618–17. 10.1371/journal.pone.0146618 26783755 PMC4718595

[B42] Feng Z. Hu W. Teresky A. K. Hernando E. Cordon-Cardo C. Levine A. J. (2007). Declining P53 Function in the Aging Process: A Possible Mechanism for the Increased Tumor Incidence in Older Populations. Proc. Natl. Acad. Sci. 104, 16633–16638. 10.1073/pnas.0708043104 17921246 PMC2034252

[B43] Franceschi C. Campisi J. (2014). Chronic Inflammation (Inflammaging) and its Potential Contribution to Age-Associated Diseases. Journals Gerontol. Ser. A: Biol. Sci. Med. Sci. 69, S4–S9. 10.1093/gerona/glu057S9 ( 24833586

[B44] Funakoshi M. Tsuda M. Muramatsu K. Hatsuda H. Morishita S. Aigaki T. (2011). A Gain-Of-Function Screen Identifies Wdb and Lkb1 as Lifespan-Extending Genes in Drosophila. Biochem. Biophysical Res. Commun. 405, 667–672. 10.1016/j.bbrc.2011.01.090 21281604

[B45] Golovko M. Y. (2005). Α-Synuclein Gene Deletion Decreases Brain Palmitate Uptake and Alters the Palmitate Metabolism in the Absence of Α-Synuclein Palmitate Binding. Biochemistry 44, 8251–8259. 10.1021/bi0502137 15938614

[B46] Gómez-Muñoz A. (2005). Ceramide-1-phosphate Promotes Cell Survival through Activation of the Phosphatidylinositol 3-kinase/protein Kinase B Pathway. FEBS Lett. 579, 3744–3750. 15978590 10.1016/j.febslet.2005.05.067

[B47] Green C. Mitchell S. Speakman J. (2017). Energy Balance and the Sphingosine-1-Phosphate/Ceramide Axis. Aging 9, 2463–2464. 10.18632/aging.101347 29242408 PMC5764382

[B48] Grimm M. O. W. (2005). Regulation of Cholesterol and Sphingomyelin Metabolism by Amyloid-β and Presenilin. Nat. Cell Biol. 7, 1118–1123. 10.1038/ncb1313 16227967

[B49] Hait N. C. Allegood J. Maceyka M. Strub G. M. Harikumar K. B. Singh S. K. (2009). Regulation of Histone Acetylation in the Nucleus by Sphingosine-1-Phosphate. Science 325, 1254–1257. 10.1126/science.1176709 19729656 PMC2850596

[B50] Halbleib K. Pesek K. Covino R. Hofbauer H. F. Wunnicke D. Hänelt I. (2017). Activation of the Unfolded Protein Response by Lipid Bilayer Stress. Mol. Cell 67, 673e8–684. 10.1016/j.molcel.2017.06.012 28689662

[B51] Hammad S. M. (2020). Transcriptomics Reveal Altered Metabolic and Signaling Pathways in Podocytes Exposed to C16. Genes (Basel) 11, 178. 10.3390/genes11020178 PMC7073971 32045989

[B52] Hanahan D. Weinberg R. A. (2011). Hallmarks of Cancer: The Next Generation. Cell 144, 646–674. 10.1016/j.cell.2011.02.013 21376230

[B53] Haughey N. J. (2010). Sphingolipids in Neurodegeneration. Neuromol. Med. 12, 301–305. 10.1007/s12017-010-8135-5 PMC4083244 20737248

[B54] He Q. Wang G. Wakade S. Dasgupta S. Dinkins M. Kong J. N. (2014). Primary Cilia in Stem Cells and Neural Progenitors Are Regulated by Neutral Sphingomyelinase 2 and Ceramide. MBoC 25, 1715–1729. 10.1091/mbc.e13-12-0730 24694597 PMC4038499

[B55] Heffernan-Striud L. Obeid L. M. (2013). Sphingosine Kinase 1 in Cancer. Adv. Cancer Res. 117, 201–235. 23290781 10.1016/B978-0-12-394274-6.00007-8 PMC5491387

[B56] Hernandez D. G. Nalls M. A. Gibbs J. R. Arepalli S. van der Brug M. Chong S. (2011). Distinct DNA Methylation Changes Highly Correlated with Chronological Age in the Human Brain. Hum. Mol. Genet. 20, 1164–1172. 10.1093/hmg/ddq561 21216877 PMC3043665

[B57] Hetz C. (2012). The Unfolded Protein Response: Controlling Cell Fate Decisions under ER Stress and beyond. Nat. Rev. Mol. Cell Biol. 13, 89–102. 10.1038/nrm3270 22251901

[B58] Hicks D. A. Nalivaeva N. N. Turner A. J. (2012). Lipid Rafts and Alzheimer's Disease: Protein-Lipid Interactions and Perturbation of Signaling. Front. Physio. 3 (JUN), 189–218. 10.3389/fphys.2012.00189 PMC3381238 22737128

[B59] Hirabayashi Y. Furuya S. (2008). Roles of L-Serine and Sphingolipid Synthesis in Brain Development and Neuronal Survival. Prog. Lipid Res. 47, 188–203. 10.1016/j.plipres.2008.01.003 18319065

[B60] Hirosha Geekiyanagea E. Upadhyeb Aditi. Chan C. (2013). Inhibition of SPT Reduces Aβ and Tau Hyperphosphorylation in a Mouse Model, a Safe Therapeutic Strategy for Alzheimer’s Disease. Neurobiol. Aging 34, 2037–2051. 10.1016/j.neurobiolaging.2013.02.001 23528227 PMC3651806

[B61] Hong S. Huo H. Xu J. Liao K. (2004). Insulin-like Growth Factor-1 Receptor Signaling in 3T3-L1 Adipocyte Differentiation Requires Lipid Rafts but Not Caveolae. Cell Death Differ. 11, 714–723. 10.1038/sj.cdd.4401405 15002041

[B62] Horvath S. Raj K. (2018). DNA Methylation-Based Biomarkers and the Epigenetic Clock Theory of Ageing. Nat. Rev. Genet. 19, 371–384. 10.1038/s41576-018-0004-3 29643443

[B63] Inoki K. Zhu T. Guan K.-L. (2003). TSC2 Mediates Cellular Energy Response to Control Cell Growth and Survival. Cell 115, 577–590. 10.1016/s0092-8674(03)00929-2 14651849

[B64] Jana1 A. Hogan2 E. L. Pahan K. (2009). Ceramide and Neurodegeneration: Susceptibility of Neurons and Oligodendrocytes to Cell Damage and Death Arundhati. J. Neurol. Sci. 278, 5–15. 10.1016/j.jns.2008.12.010 19147160 PMC2660887

[B65] Jensen J.-M. Forl M. Winoto-Morbach S. Seite S. Schunck M. Proksch E. (2005). Acid and Neutral Sphingomyelinase, Ceramide Synthase, and Acid Ceramidase Activities in Cutaneous Aging. Exp. Dermatol. 14, 609–618. 10.1111/j.0906-6705.2005.00342.x 16026583

[B66] Jęśko H. Stępień A. Lukiw W. J. Strosznajder R. P. (2019). The Cross-Talk between Sphingolipids and Insulin-like Growth Factor Signaling: Significance for Aging and Neurodegeneration. Mol. Neurobiol. 56, 3501–3521. 10.1007/s12035-018-1286-3 30140974 PMC6476865

[B67] Johnson K. R. (2003). Role of Human Sphingosine-1-Phosphate Phosphatase 1 in the Regulation of Intra- and Extracellular Sphingosine-1-Phosphate Levels and Cell Viability. J. Biol. Chem. 278, 34541–34547. 10.1074/jbc.m301741200 12815058

[B68] Ju T. F. Gao D. Q. Fang Z. Y. (2016). Targeting Colorectal Cancer Cells by a Novel Sphingosine Kinase 1 Inhibitor PF-543. Biochem. Biophys. Res. Commun. 470, 728–734. 10.1016/j.bbrc.2016.01.053 26775841

[B69] Kapitonov D. (2010). Targeting Sphingosine Kinase 1 Inhibits AKT Signaling, Induces Apoptosis, and Suppresses Growth of Human Glioblastoma Cells and Xenografts. Cancer Res. 69, 6915–6923. 10.1158/0008-5472.CAN-09-0664 PMC2752891 19723667

[B70] Katsel P. Li C. Haroutunian V. (2007). Gene Expression Alterations in the Sphingolipid Metabolism Pathways during Progression of Dementia and Alzheimer’s Disease: A Shift toward Ceramide Accumulation at the Earliest Recognizable Stages of Alzheimer’s Disease? Neurochem. Res. 32, 845–856. 10.1007/s11064-007-9297-x 17342407

[B71] Kaushik S. Cuervo A. M. (2015). Proteostasis and Aging. Nat. Med. 21, 1406–1415. 10.1038/nm.4001 26646497

[B72] Kawamori T. (2009). Role for Sphingosine Kinase 1 in colon Carcinogenesis. FASEB J. 23, 405–414. 10.1096/fj.08-117572 18824518 PMC2630788

[B73] Kennedy P. C. (2011). Characterization of a Sphingosine 1-phosphate Receptor Antagonist Prodrug. J. Pharmacol. Exp. Ther. 338, 879–889. 10.1124/jpet.111.181552 21632869 PMC3164350

[B74] Kenyon C. J. (2010). The Genetics of Ageing. Nature 464, 504–512. 10.1038/nature08980 20336132

[B75] Khan A. H. Zou Z. Xiang Y. Chen S. Tian X.-L. (2019). Conserved Signaling Pathways Genetically Associated with Longevity across the Species. Biochim. Biophys. Acta (BBA) - Mol. Basis Dis. 1865, 1745–1755. 10.1016/j.bbadis.2018.09.001 31109448

[B76] Kim H.-E. Grant A. R. Simic M. S. Kohnz R. A. Nomura D. K. Durieux J. (2016). Lipid Biosynthesis Coordinates a Mitochondrial-To-Cytosolic Stress Response. Cell 166, 1539e16–1552. 10.1016/j.cell.2016.08.027 27610574 PMC5922983

[B77] Kim M. K. Lee W. Yoon G.-H. Chang E.-J. Choi S.-C. Kim S. W. (2019). Links between Accelerated Replicative Cellular Senescence and Down-Regulation of SPHK1 Transcription. BMB Rep. 52, 220–225. 10.5483/bmbrep.2019.52.3.012 30885289 PMC6476483

[B78] Kim M. Park J. W. Lee E. J. Kim S. Shin S. H. Ahn J. H. (2018). C16-ceramide and sphingosine�1-phosphate/S1PR2 H-ave O-pposite E-ffects on C-ell G-rowth through mTOR S-ignaling P-athway R-egulation. Oncol. Rep. 40, 2977–2987. 10.3892/or.2018.6689 30226616

[B79] Kim Y. E. Hipp M. S. Bracher A. Hayer-Hartl M. Ulrich Hartl F. (2013). Molecular Chaperone Functions in Protein Folding and Proteostasis. Annu. Rev. Biochem. 82, 323–355. 10.1146/annurev-biochem-060208-092442 23746257

[B80] Kolter T. Sandhoff K. (2005). Principles of Lysosomal Membrane Digestion: Stimulation of Sphingolipid Degradation by Sphingolipid Activator Proteins and Anionic Lysosomal Lipids. Annu. Rev. Cell Dev. Biol. 21, 81–103. 10.1146/annurev.cellbio.21.122303.120013 16212488

[B81] Kong J. N. Hardin K. Dinkins M. Wang G. He Q. Mujadzic T. (2015). Regulation of Chlamydomonas Flagella and Ependymal Cell Motile Cilia by Ceramide-Mediated Translocation of GSK3. MBoC 26, 4451–4465. 10.1091/mbc.e15-06-0371 26446842 PMC4666139

[B82] Kraveka J. M. Li L. Szulc Z. M. Bielawski J. Ogretmen B. Hannun Y. A. (2007). Involvement of Dihydroceramide Desaturase in Cell Cycle Progression in Human Neuroblastoma Cells. J. Biol. Chem. 282, 16718–16728. 10.1074/jbc.m700647200 17283068 PMC2084375

[B83] Kurano M. Tsukamoto K. Hara M. Ohkawa R. Ikeda H. Yatomi Y. (2015). LDL Receptor and ApoE Are Involved in the Clearance of ApoM-Associated Sphingosine 1-phosphate. J. Biol. Chem. 290, 2477–2488. 10.1074/jbc.m114.596445 25505264 PMC4303696

[B84] Kurtishi A. Rosen B. Patil K. S. Alves G. W. Møller S. G. (2019). Cellular Proteostasis in Neurodegeneration. Mol. Neurobiol. 56, 3676–3689. 10.1007/s12035-018-1334-z 30182337

[B85] Lankadasari M. B. (2018). Targeting S1PR1/STAT3 Loop Abrogates Desmoplasia and Chemosensitizes Pancreatic Cancer to Gemcitabine. Theranostics 8, 3824–3840. 10.7150/thno.25308 30083262 PMC6071521

[B86] Law B. A. Liao X. Moore K. S. Southard A. Roddy P. Ji R. (2018). Lipotoxic Very‐long‐chain Ceramides Cause Mitochondrial Dysfunction, Oxidative Stress, and Cell Death in Cardiomyocytes. FASEB J. 32, 1403–1416. 10.1096/fj.201700300r 29127192 PMC5892719

[B87] Lépine S. Allegood J. C. Edmonds Y. Milstien S. Spiegel S. (2011). Autophagy Induced by Deficiency of Sphingosine-1-Phosphate Phosphohydrolase 1 Is Switched to Apoptosis by Calpain-Mediated Autophagy-Related Gene 5 (Atg5) Cleavage. J. Biol. Chem. 286, 44380–44390. 10.1074/jbc.M111.257519 22052905 PMC3247960

[B88] Li M-H. Swenson R. Harel M. Jana S. Stolarzewicz E. Shapiro L. H. (2015). Antitumor Activity of a Novel Sphingosine-1-Phosphate 2 Antagonist, AB1, in Neuroblastoma. J. Pharm. Sci. 28, 1887–1890. 10.1124/jpet.115.224519 PMC4538871 26105954

[B89] Lin G. (2018). Phospholipase PLA2G6, a Parkinsonism-Associated Gene, Affects Vps26 and Vps35, Retromer Function, and Ceramide Levels, Similar to α-Synuclein Gain. Cell Metab 28, 605–618. 10.1016/j.cmet.2018.05.019 29909971

[B90] Lithgow G. J. Walker G. A. (2002). Stress Resistance as a Determinate of *C. elegans* Lifespan. Mech. Ageing Development 123, 765–771. 10.1016/s0047-6374(01)00422-5 11869734

[B91] Liu C.-C. Kanekiyo T. Xu H. Bu G. Bu G. (2013). Apolipoprotein E and Alzheimer Disease: Risk, Mechanisms and Therapy. Nat. Rev. Neurol. 9, 106–118. 10.1038/nrneurol.2012.263 23296339 PMC3726719

[B92] Liu J. Huang X. Withers B. R. Blalock E. Liu K. Dickson R. C. (2013). Reducing Sphingolipid Synthesis Orchestrates Global Changes to Extend Yeast Lifespan. Aging Cell 12, 833–841. 10.1111/acel.12107 23725375 PMC3773046

[B93] Ljubicic V. Hood D. A. (2009). Diminished Contraction-Induced Intracellular Signaling towards Mitochondrial Biogenesis in Aged Skeletal Muscle. Aging Cell 8, 394–404. 10.1111/j.1474-9726.2009.00483.x 19416128

[B94] Llewellyn K. J. Nalbandian A. Jung K.-M. Nguyen C. Avanesian A. Mozaffar T. (2014). Lipid-enriched Diet Rescues Lethality and Slows Down Progression in a Murine Model of VCP-Associated Disease. Hum. Mol. Genet. 23, 1333–1344. 10.1093/hmg/ddt523 24158850 PMC3919004

[B95] López-Otín C. Blasco M. A. Partridge L. Serrano M. Kroemer G. (2013). The Hallmarks of Aging. Cell 153, 1194–1217. 10.1016/j.cell.2013.05.039 23746838 PMC3836174

[B96] Lu H. Yuan H. Chen S. Huang L. Xiang H. Yang G. (2012). Senescent Endothelial Dysfunction Is Attributed to the Up-Regulation of Sphingosine-1-Phosphate Receptor-2 in Aged Rats. Mol. Cell. Biochem. 363, 217–224. 10.1007/s11010-011-1173-y 22139303

[B97] Madeo F. Carmona-Gutierrez D. Hofer S. J. Kroemer G. (2019). Caloric Restriction Mimetics against Age-Associated Disease: Targets, Mechanisms, and Therapeutic Potential. Cell Metab. 29, 592–610. 10.1016/j.cmet.2019.01.018 30840912

[B98] Maeng H. J. Song J.-H. Kim G.-T. Song Y.-J. Lee K. Kim J.-Y. (2017). Celecoxib-mediated Activation of Endoplasmic Reticulum Stress Induces De Novo Ceramide Biosynthesis and Apoptosis in Hepatoma HepG2 Cells. BMB Rep. 50, 144–149. 10.5483/bmbrep.2017.50.3.197 28193314 PMC5422027

[B99] Maeurer C. Holland S. Pierre S. Potstada W. Scholich K. (2009). Sphingosine-1-phosphate Induced mTOR-Activation Is Mediated by the E3-Ubiquitin Ligase PAM. Cell Signal. 21, 293–300. 10.1016/j.cellsig.2008.10.016 19000755

[B100] Mahdy A. E. M. (2009). Acid Ceramidase Upregulation in Prostate Cancer Cells Confers Resistance to Radiation: AC Inhibition, a Potential Radiosensitizer. Mol. Ther. 17, 430–438. 10.1038/mt.2008.281 19107118 PMC2835081

[B101] Malaplate-Armand C. (2006). Soluble Oligomers of Amyloid-β Peptide Induce Neuronal Apoptosis by Activating a cPLA2-dependent Sphingomyelinase-Ceramide Pathway. Neurobiol. Dis. 23, 178–189. 10.1016/j.nbd.2006.02.010 16626961

[B102] Martin J. L. Lin M. Z. McGowan E. M. Baxter R. C. (2009). Potentiation of Growth Factor Signaling by Insulin-like Growth Factor-Binding Protein-3 in Breast Epithelial Cells Requires Sphingosine Kinase Activity. J. Biol. Chem. 284, 25542–25552. 10.1074/jbc.m109.007120 19633297 PMC2757955

[B103] Martínez G. Duran‐Aniotz C. Cabral‐Miranda F. Vivar J. P. Hetz C. (2017). Endoplasmic Reticulum Proteostasis Impairment in Aging. Aging Cell 16, 615–623. 10.1111/acel.12599 28436203 PMC5506418

[B104] Mazzulli J. R. (2011). Gaucher Disease Glucocerebrosidase and α-synuclein Form a Bidirectional Pathogenic Loop in Synucleinopathies. Cell 146, 37–52. 10.1016/j.cell.2011.06.001 21700325 PMC3132082

[B105] McNaughton M. Pitman M. Pitson S. M. Pyne N. J. Pyne S. (2016). Proteasomal Degradation of Sphingosine Kinase 1 and Inhibition of Dihydroceramide Desaturase by the Sphingosine Kinase Inhibitors, SKi or ABC294640, Induces Growth Arrest in Androgen-independent LNCaP-AI Prostate Cancer Cells. Oncotarget 7, 16663–16675. 10.18632/oncotarget.7693 26934645 PMC4941342

[B106] Meacci E. Vasta V. Neri S. Farnararo M. Bruni P. (1996). Activation of Phospholipase D in Human Fibroblasts by Ceramide and Sphingosine: Evaluation of Their Modulatory Role in Bradykinin Stimulation of Phospholipase D. Biochem. Biophysical Res. Commun. 225, 392–399. 10.1006/bbrc.1996.1185 8753774

[B107] Mesa-Herrera F. Taoro-González L. Valdés-Baizabal C. Diaz M. Marín R. (2019). Lipid and Lipid Raft Alteration in Aging and Neurodegenerative Diseases: A Window for the Development of New Biomarkers. Ijms 20, 3810. 10.3390/ijms20153810 PMC6696273 31382686

[B108] Mielke M. M. Haughey N. J. (2012). Could Plasma Sphingolipids Be Diagnostic or Prognostic Biomarkers for Alzheimer’s Disease? Clin. Lipidol. 7, 525–536. 10.2217/clp.12.59 23606909 PMC3627378

[B109] Miguez A. Barriga G. G. Brito V. Straccia M. Giralt A. Ginés S. (2015). Fingolimod (FTY720) Enhances Hippocampal Synaptic Plasticity and Memory in Huntington’s Disease by Preventing p75NTR Up-Regulation and Astrocyte-Mediated Inflammation Andrés. 24(17), 4958–4970. 10.1093/hmg/ddv218 26063761

[B110] Mollinedo F. Gajate C. (2015). Lipid Rafts as Major Platforms for Signaling Regulation in Cancer. Adv. Biol. Regul. 57, 130–146. 10.1016/j.jbior.2014.10.003 25465296

[B111] Monette J. S. Gómez L. A. Moreau R. F. Dunn K. C. Butler J. A. Finlay L. A. (2011). (R)-α-Lipoic Acid Treatment Restores Ceramide Balance in Aging Rat Cardiac Mitochondria. Pharmacol. Res. 63, 23–29. 10.1016/j.phrs.2010.09.007 20934512 PMC3268156

[B112] Montoliu I. (2014). Serum Profiling of Healthy Aging Identifies Phospho- and Sphingolipid Species as Markers of Human Longevity. Aging (Albany. NY) 6, 9–25. 10.18632/aging.100630 24457528 PMC3927806

[B113] Morad S. A. F. Cabot M. C. (2013). Ceramide-orchestrated Signalling in Cancer Cells. Nat. Rev. Cancer 13, 51–65. 10.1038/nrc3398 23235911

[B114] Mu C. (2013). Sphingolipids in Obesity, Type 2 Diabetes, and Metabolic Disease. Sphingolipids Dis. 216, 247–264.

[B115] Munk R. Anerillas C. Rossi M. Tsitsipatis D. Martindale J. L. Herman A. B. (2021). Acid Ceramidase Promotes Senescent Cell Survival. Aging 13, 15750–15769. 10.18632/aging.203170 34102611 PMC8266329

[B116] Muñoz-Espín D. (2013). Programmed Cell Senescence during Mammalian Embryonic Development. Cell 155, 1104. 24238962 10.1016/j.cell.2013.10.019

[B117] Nadon N. L. Strong R. Miller R. A. Harrison D. E. (2017). NIA Interventions Testing Program: Investigating Putative Aging Intervention Agents in a Genetically Heterogeneous Mouse Model. EBioMedicine 21, 3–4. 10.1016/j.ebiom.2016.11.038 27923560 PMC5514387

[B118] Nelson G. Wordsworth J. Wang C. Jurk D. Lawless C. Martin‐Ruiz C. (2012). A Senescent Cell Bystander Effect: Senescence‐induced Senescence. Aging Cell 11, 345–349. 10.1111/j.1474-9726.2012.00795.x 22321662 PMC3488292

[B119] Neviani P. (2007). FTY720, a New Alternative for Treating Blast Crisis Chronic Myelogenous Leukemia and Philadelphia Chromosome-Positive Acute Lymphocytic Leukemia. J. Clin. Invest. 117, 2408–2421. 10.1172/jci31095 17717597 PMC1950458

[B120] Newton J. Lima S. Maceyka M. Spiegel S. (2015). Revisiting the Sphingolipid Rheostat: Evolving Concepts in Cancer Therapy. Exp. Cell Res. 333, 195–200. 10.1016/j.yexcr.2015.02.025 25770011 PMC4415605

[B121] Nguyen-Tran D.-H. Hait N. C. Sperber H. Qi J. Fischer K. Ieronimakis N. (2014). Molecular Mechanism of Sphingosine-1-Phosphate Action in Duchenne Muscular Dystrophy. Dis. Model. Mech. 7, 41–54. 10.1242/dmm.013631 24077965 PMC3882047

[B122] Niccoli T. Partridge L. (2012). Ageing as a Risk Factor for Disease. Curr. Biol. 22, R741–R752. 10.1016/j.cub.2012.07.024 22975005

[B123] Nishizuka Y. (1992). Intracellular Signaling by Hydrolysis of Phospholipids and Activation of Protein Kinase C. Science 258, 607–614. 10.1126/science.1411571 1411571

[B124] Nuss J. E. Choksi K. B. DeFord J. H. Papaconstantinou J. (2008). Decreased Enzyme Activities of Chaperones PDI and BiP in Aged Mouse Livers. Biochem. Biophysical Res. Commun. 365, 355–361. 10.1016/j.bbrc.2007.10.194 PMC2238339 17996725

[B125] Ohno-Iwashita Y. Shimada Y. Hayashi M. Inomata M. (2010). Plasma Membrane Microdomains in Aging and Disease. Geriatr. Gerontol. Int. 10, S41–S52. 10.1111/j.1447-0594.2010.00600.x 20590841

[B126] Oskouian B. (2006). Sphingosine-1-phosphate Lyase Potentiates Apoptosis via P53- and P38-dependent Pathways and Is Down-Regulated in colon Cancer. Proc. Natl. Acad. Sci. U. S. A. 103, 17384–17389. 10.1073/pnas.0600050103 17090686 PMC1859938

[B127] Pan T. Kondo S. Le W. Jankovic J. (2008). The Role of Autophagy-Lysosome Pathway in Neurodegeneration Associated with Parkinson’s Disease. Brain 131, 1969–1978. 10.1093/brain/awm318 18187492

[B128] Panchal M. (2014). Ceramides and Sphingomyelinases in Senile Plaques. Neurobiol. Dis. 65, 193–201. 10.1016/j.nbd.2014.01.010 24486621

[B129] Panneer Selvam S. De Palma R. M. Oaks J. J. Oleinik N. Peterson Y. K. Stahelin R. V. (2015). Binding of the Sphingolipid S1P to hTERT Stabilizes Telomerase at the Nuclear Periphery by Allosterically Mimicking Protein Phosphorylation. Sci. Signal. 8, ra58. 10.1126/scisignal.aaa4998 26082434 PMC4492107

[B130] Pardo A. D. Amico E. Favellato M. Castrataro R. Fucile S. Squitieri F. (2014). FTY720 ( Fingolimod ) Is a Neuroprotective and Disease-Modifying Agent in Cellular and Mouse Models of Huntington Disease. Hum. Mol. Genet. 23, 2251–2265. 10.1093/hmg/ddt615 24301680

[B131] Pardo A. D. Basit A. Armirotti A. Amico E. Castaldo S. Pepe G. (2017). De Novo Synthesis of Sphingolipids Is Defective in Experimental Models of Huntington’s Disease. Front. Neurosci. 11, 1–10. 10.3389/fnins.2017.00698 29311779 PMC5742211

[B132] Pardo A. D. Castaldo S. Amico E. Pepe G. Marracino F. Capocci L. (2018). Stimulation of S1PR 5 with A-971432, a Selective Agonist, Preserves Blood – Brain Barrier Integrity and Exerts Therapeutic Effect in an Animal Model of Huntington’s Disease. Hum. Mol. Genet. 27, 2490–2501. 10.1093/hmg/ddy153 29688337

[B133] Pardo A. D. (2017). Defective Sphingosine-1-Phosphate Metabolism is a Druggable Target in Huntington’s Disease. Scientific Rep. 7, 1–14. 10.1038/s41598-017-05709-y PMC5509685 28706199

[B134] Pardo A. D. (2020). Neurobiology of Disease Treatment with K6PC-5, a Selective Stimulator of SPHK1, Ameliorates Intestinal Homeostasis in an Animal Model of Huntington’s Disease. Neurobiol. Dis. 143, 105009. 10.1016/j.nbd.2020.105009 32634578

[B135] Perks C. M. Bowen S. Gill Z. P. Newcomb P. V. Holly J. M. P. (1999). Differential IGF-independent Effects of Insulin-like Growth Factor Binding Proteins (1-6) on Apoptosis of Breast Epithelial Cells. J. Cell. Biochem. 75, 652–664. 10.1002/(sici)1097-4644(19991215)75:4<652:aid-jcb11>3.0.co;2-0 10572248

[B136] Perry D. K. (2002). Serine Palmitoyltransferase: Role in Apoptotic De Novo Ceramide Synthesis and Other Stress Responses. Biochim. Biophys. Acta (BBA) - Mol. Cell Biol. Lipids 1585, 146–152. 10.1016/s1388-1981(02)00335-9 12531548

[B137] Pewzner-Jung Y. Ben-Dor S. Futerman A. H. (2006). When Do Lasses (Longevity Assurance Genes) Become CerS (Ceramide Synthases)? J. Biol. Chem. 281, 25001–25005. 10.1074/jbc.r600010200 16793762

[B138] Phillips D. C. Hunt J. T. Moneypenny C. G. Maclean K. H. McKenzie P. P. Harris L. C. (2007). Ceramide-induced G2 Arrest in Rhabdomyosarcoma (RMS) Cells Requires p21Cip1/Waf1 Induction and Is Prevented by MDM2 Overexpression. Cell Death Differ 14, 1780–1791. 10.1038/sj.cdd.4402198 17627285

[B139] Pohl C. Dikic I. (2019). Cellular Quality Control by the Ubiquitin-Proteasome System and Autophagy. Science 366, 818–822. 10.1126/science.aax3769 31727826

[B140] Proia R. L. Hla T. (2015). Emerging Biology of Sphingosine-1-Phosphate: Its Role in Pathogenesis and Therapy. J. Clin. Invest. 125, 1379–1387. 10.1172/jci76369 25831442 PMC4409021

[B141] Puglielli L. Ellis B. C. Saunders A. J. Kovacs D. M. (2003). Ceramide Stabilizes β-Site Amyloid Precursor Protein-Cleaving Enzyme 1 and Promotes Amyloid β-Peptide Biogenesis. J. Biol. Chem. 278, 19777–19783. 10.1074/jbc.m300466200 12649271

[B142] Pyne N. J. McNaughton M. Boomkamp S. MacRitchie N. Evangelisti C. Martelli A. M. (2016). Role of Sphingosine 1-phosphate Receptors, Sphingosine Kinases and Sphingosine in Cancer and Inflammation. Adv. Biol. Regul. 60, 151–159. 10.1016/j.jbior.2015.09.001 26429117

[B143] Pyszko J. A. Strosznajder J. B. (2014). The Key Role of Sphingosine Kinases in the Molecular Mechanism of Neuronal Cell Survival and Death in an Experimental Model of Parkinson’s Disease. Folia Neuropathol. 52, 260–269. 10.5114/fn.2014.45567 25310737

[B144] Rea I. M. Gibson D. S. McGilligan V. McNerlan S. E. Alexander H. D. Ross O. A. (2018). Age and Age-Related Diseases: Role of Inflammation Triggers and Cytokines. Front. Immunol. 9, 1–28. 10.3389/fimmu.2018.00586 29686666 PMC5900450

[B145] Realini N. (2016). Acid Ceramidase in Melanoma: Expression, Localization, and Effects of Pharmacological Inhibition. J. Biol. Chem. 291, 2422–2434. 10.1074/jbc.m115.666909 26553872 PMC4732224

[B146] Reznick R. M. Zong H. Li J. Morino K. Moore I. K. Yu H. J. (2007). Aging-Associated Reductions in AMP-Activated Protein Kinase Activity and Mitochondrial Biogenesis. Cell Metab. 5, 151–156. 10.1016/j.cmet.2007.01.008 17276357 PMC1885964

[B147] Riebeling C. Allegood J. C. Wang E. Merrill A. H. Futerman A. H. (2003). Two Mammalian Longevity Assurance Gene (LAG1) Family Members, Trh1 and Trh4, Regulate Dihydroceramide Synthesis Using Different Fatty Acyl-CoA Donors. J. Biol. Chem. 278, 43452–43459. 10.1074/jbc.m307104200 12912983

[B148] Rivas D. A. Morris E. P. Haran P. H. Pasha E. P. Morais M. d. S. Dolnikowski G. G. (2012). Increased Ceramide Content and NFκB Signaling May Contribute to the Attenuation of Anabolic Signaling after Resistance Exercise in Aged Males. J. Appl. Physiol. 113, 1727–1736. 10.1152/japplphysiol.00412.2012 23042913 PMC3774074

[B149] Robert J. Passos J. F. Khosla S. Tamara Tchkonia J. L. K. (2020). Reducing Senescent Cell Burden in Aging and Disease Robert. Trends. Mol. Med. 26, 630–638. 32589933 10.1016/j.molmed.2020.03.005 PMC7857028

[B150] Rocha E. M. (2015). Sustained Systemic Glucocerebrosidase Inhibition Induces Brain α-Synuclein Aggregation, Microglia and Complement C1q Activation in Mice. Antioxid. Redox Signal 23, 550–564. 10.1089/ars.2015.6307 26094487 PMC4544823

[B151] Rodgers A. Mormeneo D. Long J. S. Delgado A. Pyne N. J. Pyne S. (2009). Sphingosine 1-phosphate Regulation of Extracellular Signal-Regulated Kinase-1/2 in Embryonic Stem Cells. Stem Cell Development 18, 1319–1330. 10.1089/scd.2009.0023 19228106

[B152] Sawamura N. Ko M. Yu W. Zou K. Hanada K. Suzuki T. (2004). Modulation of Amyloid Precursor Protein Cleavage by Cellular Sphingolipids. J. Biol. Chem. 279, 11984–11991. 10.1074/jbc.m309832200 14715666

[B153] Schoenauer R. Larpin Y. Babiychuk E. B. Drücker P. Babiychuk V. S. Avota E. (2019). Down‐regulation of Acid Sphingomyelinase and Neutral Sphingomyelinase‐2 Inversely Determines the Cellular Resistance to Plasmalemmal Injury by Pore‐forming Toxins. FASEB J. 33, 275–285. 10.1096/fj.201800033r 29979630

[B154] Schreiber K. H. (2019). A Novel Rapamycin Analog Is Highly Selective for mTORC1 *In Vivo* . Nat. Commun. 10, 1–12. 10.1038/s41467-019-11174-0 31324799 PMC6642166

[B155] Sheng M. C. M. L. (2018). Sphingolipids and Their Metabolism in Psysiology and Disease. Nat. Rev. Mol. Cell Biol. 19, 175–191. 29165427 10.1038/nrm.2017.107 PMC5902181

[B156] Sidransky E. Nalls M. A. Aasly J. O. Aharon-Peretz J. Annesi G. Barbosa E. R. (2010). Multi-center Analysis of Glucocerebrosidase Mutations in Parkinson Disease. N. Engl. J. Med. 361, 1651–1661. 10.1056/NEJMoa0901281 PMC2856322 19846850

[B157] Simons M. Keller P. De Strooper B. Beyreuther K. Dotti C. G. Simons K. (1998). Cholesterol Depletion Inhibits the Generation of -amyloid in Hippocampal Neurons. Proc. Natl. Acad. Sci. 95, 6460–6464. 10.1073/pnas.95.11.6460 9600988 PMC27798

[B158] Solana R. Tarazona R. Gayoso I. Lesur O. Dupuis G. Fulop T. (2012). Innate Immunosenescence: Effect of Aging on Cells and Receptors of the Innate Immune System in Humans. Semin. Immunol. 24, 331–341. 10.1016/j.smim.2012.04.008 22560929

[B159] Song P. Trajkovic K. Tsunemi T. Krainc D. (2016). Parkin Modulates Endosomal Organization and Function of the Endo-Lysosomal Pathway. J. Neurosci. 36, 2425–2437. 10.1523/jneurosci.2569-15.2016 26911690 PMC6705497

[B160] Sugimura R. Li L. (2010). Noncanonical Wnt Signaling in Vertebrate Development, Stem Cells, and Diseases. Birth Defects Res. C: Embryo Today Rev. 90, 243–256. 10.1002/bdrc.20195 21181886

[B161] Sugiura M. Kono K. Liu H. Shimizugawa T. Minekura H. Spiegel S. (2002). Ceramide Kinase, a Novel Lipid Kinase. J. Biol. Chem. 277, 23294–23300. 10.1074/jbc.m201535200 11956206

[B162] Suh Y. Atzmon G. Cho M.-O. Hwang D. Liu B. Leahy D. J. (2008). Functionally Significant Insulin-like Growth Factor I Receptor Mutations in Centenarians. Proc. Natl. Acad. Sci. 105, 3438–3442. 10.1073/pnas.0705467105 18316725 PMC2265137

[B163] Susan P. (2005). ER Stress-Induced Cell Death Mechanisms Renata. Bone 23, 1–7.

[B164] Szymiczek A. (2017). FTY720 Inhibits Mesothelioma Growth *In Vitro* and in a Syngeneic Mouse Model. J. Transl. Med. 15, 1–11. 10.1186/s12967-017-1158-z 28298211 PMC5353897

[B165] Taha T. A. Osta W. Kozhaya L. Bielawski J. Johnson K. R. Gillanders W. E. (2004). Down-regulation of Sphingosine Kinase-1 by DNA Damage. J. Biol. Chem. 279, 20546–20554. 10.1074/jbc.m401259200 14988393

[B166] Tam A. B. Roberts L. S. Chandra V. Rivera I. G. Nomura D. K. Forbes D. J. (2018). The UPR Activator ATF6 Responds to Proteotoxic and Lipotoxic Stress by Distinct Mechanisms. Developmental Cell 46, 327e7–343. 10.1016/j.devcel.2018.04.023 30086303 PMC6467773

[B167] Tanaka K. Matsuda N. (2014). Proteostasis and Neurodegeneration: The Roles of Proteasomal Degradation and Autophagy. Biochim. Biophys. Acta (BBA) - Mol. Cell Res. 1843, 197–204. 10.1016/j.bbamcr.2013.03.012 23523933

[B168] Taylor R. C. (2016). Aging and the UPR(ER). Brain Res. 1648, 588–593. 10.1016/j.brainres.2016.04.017 27067187

[B169] Taylor R. C. Dillin A. (2013). XBP-1 Is a Cell-Nonautonomous Regulator of Stress Resistance and Longevity. Cell 153, 1435–1447. 10.1016/j.cell.2013.05.042 23791175 PMC4771415

[B170] Templeman N. M. Murphy C. T. (2018). Regulation of Reproduction and Longevity by Nutrient-Sensing Pathways. J. Cell Biol. 217, 93–106. 10.1083/jcb.201707168 29074705 PMC5748989

[B171] Tettamanti G. Bassi R. Viani P. Riboni L. (2003). Salvage Pathways in Glycosphingolipid Metabolism. Biochimie 85, 423–437. 10.1016/s0300-9084(03)00047-6 12770781

[B172] Tharyan R. G. Annibal A. Schiffer I. Laboy R. Atanassov I. Weber A. L. (2020). NFYB-1 Regulates Mitochondrial Function and Longevity via Lysosomal Prosaposin. Nat. Metab. 2, 387–396. 10.1038/s42255-020-0200-2 32694663

[B173] Tokunaga C. Yoshino K.-i. Yonezawa K. (2004). mTOR Integrates Amino Acid- and Energy-Sensing Pathways. Biochem. Biophysical Res. Commun. 313, 443–446. 10.1016/j.bbrc.2003.07.019 14684182

[B174] Toyama E. Q. Herzig S. Courchet J. Lewis T. L. Losón O. C. Hellberg K. (2016). AMP-activated Protein Kinase Mediates Mitochondrial Fission in Response to Energy Stress. Science 351, 275–281. 10.1126/science.aab4138 26816379 PMC4852862

[B175] Trayssac M. Hannun Y. A. Obeid L. M. (2018). Role of Sphingolipids in Senescence: Implication in Aging and Age-Related Diseases. J. Clin. Invest. 128, 2702–2712. 10.1172/jci97949 30108193 PMC6025964

[B176] Um S. H. Frigerio F. Watanabe M. (2004). Absence of S6K1 Protects against Age- and Diet-Induced Obesity while Enhancing Insulin Sensitivity. 431(September), 6. 10.1038/nature02866 15306821

[B177] Vaena S. Chakraborty P. Lee H. G. Janneh A. H. Kassir M. F. Beeson G. (2021). Aging-dependent Mitochondrial Dysfunction Mediated by Ceramide Signaling Inhibits Antitumor T Cell Response. Cell Rep. 35, 109076. 10.1016/j.celrep.2021.109076 33951438 PMC8127241

[B178] Van Deursen J. M. (2014). The Role of Senescent Cells in Ageing. Nature 509, 439–446. 10.1038/nature13193 24848057 PMC4214092

[B179] Varma V. R. Oommen A. M. Varma S. Casanova R. An Y. Andrews R. M. (2018). Brain and Blood Metabolite Signatures of Pathology and Progression in Alzheimer Disease: A Targeted Metabolomics Study. Plos Med. 15, e1002482. 10.1371/journal.pmed.1002482 29370177 PMC5784884

[B180] Venable M. E. Lee J. Y. Smyth M. J. Bielawska A. Obeid L. M. (1995). Role of Ceramide in Cellular Senescence. J. Biol. Chem. 270, 30701–30708. 10.1074/jbc.270.51.30701 8530509

[B181] Vermeulen C. Loeschcke V. (2007). Longevity and the Stress Response in Drosophila. Exp. Gerontol. 42, 153–159. 10.1016/j.exger.2006.09.014 17110070

[B182] Volmer R. Van Der Ploeg K. Ron D. (2013). Membrane Lipid Saturation Activates Endoplasmic Reticulum Unfolded Protein Response Transducers through Their Transmembrane Domains. Proc. Natl. Acad. Sci. 110, 4628–4633. 10.1073/pnas.1217611110 23487760 PMC3606975

[B183] Vonsattel J. DiFiglia M. (1998). Huntington Disease. J. Neuropathol. Exp. Neurol. 57, 369–385. 10.1097/00005072-199805000-00001 9596408

[B184] Wakabayashi K. Tanji K. Mori F. Takahashi H. (2007). The Lewy Body in Parkinson’s Disease: Molecules Implicated in the Formation and Degradation of α-synuclein Aggregates. Neuropathology 27, 494–506. 10.1111/j.1440-1789.2007.00803.x 18018486

[B185] Weihl C. C. Pestronk A. Kimonis V. E. (2009). Valosin-containing Protein Disease: Inclusion Body Myopathy with Paget's Disease of the Bone and Fronto-Temporal Dementia. Neuromuscul. Disord. 19, 308–315. 10.1016/j.nmd.2009.01.009 19380227 PMC2859037

[B186] Wennberg A. M. V. (2018). Plasma Sphingolipids Are Associated with Gait Parameters in the Mayo Clinic Study of Aging. Journals Gerontol. - Ser. A. Biol. Sci. Med. Sci. 73, 960–965. 10.1093/gerona/glx139 PMC6001885 28977376

[B187] Wong K. (2004). Neuropathology Provides Clues to the Pathophysiology of Gaucher Disease. Mol. Genet. Metab. 82, 192–207. 10.1016/j.ymgme.2004.04.011 15234332

[B188] Wu N. Zheng B. Shaywitz A. Dagon Y. Tower C. Bellinger G. (2013). AMPK-dependent Degradation of TXNIP upon Energy Stress Leads to Enhanced Glucose Uptake via GLUT1. Mol. Cell 49, 1167–1175. 10.1016/j.molcel.2013.01.035 23453806 PMC3615143

[B189] Zamora-Pineda J. Kumar A. Suh J. H. Zhang M. Saba J. D. (2016). Dendritic Cell Sphingosine-1-Phosphate Lyase Regulates Thymic Egress. J. Exp. Med. 213, 2773–2791. 10.1084/jem.20160287 27810923 PMC5110016

[B190] Zhao P. (2017). Neuroprotective Effects of Fingolimod in Mouse Models of Parkinson’s Disease. FASEB J. 31, 172–179. 10.1096/fj.201600751r 27671228

[B191] Zhong Y. Tian F. Ma H. Wang H. Yang W. Liu Z. (2020). FTY720 Induces Ferroptosis and Autophagy via PP2A/AMPK Pathway in Multiple Myeloma Cells. Life Sci. 260, 118077. 10.1016/j.lfs.2020.118077 32810509

